# Structural Domains of CIF3 Required for Interaction with Cytokinesis Regulatory Proteins and for Cytokinesis Initiation in Trypanosoma brucei

**DOI:** 10.1128/msphere.00047-22

**Published:** 2022-03-17

**Authors:** Yasuhiro Kurasawa, Kyu Joon Lee, Ziyin Li

**Affiliations:** a Department of Microbiology and Molecular Genetics, McGovern Medical School, University of Texas Health Science Center at Houston, Houston, Texas, USA; University at Buffalo

**Keywords:** *Trypanosoma brucei*, cytokinesis, CIF3, CIF1, CIF4, FPRC, katanin80, Polo-like kinase, katanin

## Abstract

Cytokinesis in Trypanosoma brucei occurs unidirectionally from the anterior toward the posterior through mechanisms distinct from those of its human host and is controlled by a signaling pathway comprising evolutionarily conserved and trypanosome-specific regulatory proteins. The mechanistic roles and the functional interplay of these cytokinesis regulators remain poorly understood. Here, we investigate the requirement of the structural motifs in the trypanosome-specific cytokinesis regulator CIF3 for the initiation of cytokinesis, the interaction with other cytokinesis regulators, and the recruitment of CIF3-interacting proteins to the cytokinesis initiation site. We demonstrate that the internal and C-terminal coiled-coil motifs, but not the N-terminal coiled-coil motif, of CIF3 play essential roles in cytokinesis and interact with distinct cytokinesis regulators. CIF3 interacts with TbPLK, CIF1, CIF4, and FPRC through the N-terminal and C-terminal coiled-coil motifs and with KAT80 through all three coiled-coil motifs. The C-terminal coiled-coil motif of CIF3 is required for the localization of CIF3 and all of its interacting proteins, and additionally, the internal coiled-coil motif of CIF3 is required for KAT80 localization. Conversely, all the CIF3-interacting proteins are required to maintain CIF3 at the cytokinesis initiation site at different cell cycle stages. These results demonstrate that CIF3 cooperates with multiple interacting partner proteins to promote cytokinesis in T. brucei.

**IMPORTANCE** Cytokinesis is the final stage of cell division and is regulated by a signaling pathway conserved from yeast to humans. Cytokinesis in Trypanosoma brucei, an early-branching protozoan parasite causing human sleeping sickness, is regulated by mechanisms that are distinct from those of its human host, employing a number of trypanosome-specific regulatory proteins to cooperate with evolutionarily conserved regulators. The functional interplay of these cytokinesis regulators is still poorly understood. In this work, we investigated the structural requirement of the trypanosome-specific cytokinesis regulator CIF3 for the initiation of cytokinesis, the interaction with other cytokinesis regulatory proteins, and the recruitment of CIF3-interacting proteins. We demonstrated that different structural motifs of CIF3 played distinct roles in cytokinesis, interacted with distinct cytokinesis regulatory proteins, and were required for the recruitment of distinct cytokinesis regulatory proteins. These findings provided novel insights into the cooperative roles of cytokinesis regulators in promoting cytokinesis in T. brucei.

## INTRODUCTION

Cytokinesis is the final step of cell division, and the control of cytokinesis in eukaryotes requires a complex interplay among numerous regulatory proteins that act in concert at the site of cell division and the cytoplasmic cleavage furrow (1, 2). Despite the differences in genetic complexity and cell morphology among eukaryotic organisms, the cytokinesis regulatory pathway appears to be considerably conserved across evolution from fungi to humans, which divides along the cell’s short axis, requiring actomyosin contractile-ring-mediated membrane constriction (2). Many parasitic protozoans, including Giardia lamblia, Trypanosoma brucei, Trypanosoma cruzi, and *Leishmania* spp., however, divide along the cell’s longitudinal axis without forming an actomyosin contractile ring (3). The molecular mechanisms underlying this unusual mode of cell division remain poorly understood; hence, further exploration might provide novel insights into the evolution and divergence of the cytokinesis machinery and the signaling cascade and, importantly, may discover new drug targets for chemotherapeutic treatment of the human diseases caused by infection by these parasites.

Trypanosoma brucei, a flagellated, unicellular protozoan causing sleeping sickness in humans and nagana in cattle in sub-Saharan Africa, is a well-established model organism for understanding the mechanisms of the unusual actomyosin-independent cytokinesis due to the availability of tools for genetic manipulation. The parasite has a complex life cycle, infecting an insect vector, the tsetse fly, and multiple mammalian hosts, and proliferates through binary cell fission in the fly’s midgut and the mammal’s bloodstream. A trypanosome cell contains a single flagellum, which is nucleated from the basal body located in the posterior part of the cell, exits the cell through the flagellar pocket, and extends toward the anterior tip of the cell (4). The flagellum is attached to the cell body through a specialized cytoskeletal structure termed the flagellum attachment zone (FAZ), which contains an intracellular protein filament (FAZ filament) that originates, near the flagellar pocket, from a fishhook-like cytoskeletal structure termed the hook complex and extends to the anterior cell tip (5). During the cell division cycle, the basal body is among the first duplicated cytoskeletal structures and organelles. Once a new flagellum is synthesized from the newly formed basal body, other flagellum-associated cytoskeletal structures, including the hook complex, the FAZ, and the flagellar pocket collar, and the kinetoplast (the cell’s mitochondrial DNA network) start to duplicate, followed by nuclear duplication. The newly formed flagellum and its associated FAZ extend toward the cell anterior tip. The lengths of the new flagellum and the new FAZ determine the length of the new-flagellum daughter cell (6, 7), likely by defining the cell division plane, and the distal tip of the new FAZ (or the anterior tip of the new-flagellum daughter cell) constitutes the cytokinesis initiation site from which the cleavage furrow ingresses along the division fold formed through membrane invagination between the new and the old flagella toward the nascent posterior end of the old-flagellum daughter cell (8). Cytokinesis occurs unidirectionally along the longitudinal axis from the anterior tip of the new-flagellum daughter cell toward the nascent posterior of the old-flagellum daughter cell, thereby bisecting the two daughter cells (8, 9).

The lack of a myosin II ortholog (10) and the lack of an involvement of actin in cell division in T. brucei (11) manifest the unusual mechanism of cytokinesis in this early-diverging microbial eukaryote. The signaling pathway governing cytokinesis in T. brucei, which is being delineated in recent years, comprises both evolutionarily conserved and trypanosome-specific regulatory proteins that act in concert at the distal tip of the new FAZ and the cleavage furrow to drive cytokinesis initiation and progression (9, 12–24). The two evolutionarily conserved protein kinases, the Polo-like kinase TbPLK (T. brucei Polo-Like Kinase) and the aurora B kinase TbAUK1 (T. brucei Aurora Kinase 1), are both required for cytokinesis (12–15), and they act sequentially at the new FAZ tip and cooperate with the trypanosome-specific cytokinesis regulator CIF1 (Cytokinesis Initiation Factor 1) to promote cytokinesis initiation (19). CIF1 appears to function as an orchestrator of cytokinesis initiation by interacting and cooperating with multiple cytokinesis regulators, including the trypanosome-specific proteins CIF2, CIF3, CIF4, FPRC (FAZ tip Protein Required for Cytokinesis), KPP1 (Kinetoplastid-specific Protein Phosphatase 1), KLIF (Kinesin-Localized to the Ingressing Furrow), and FRW1 (Furrow 1) and a katanin80 homolog named KAT80 (18, 20–23, 25). Although these proteins play essential roles in promoting cytokinesis in T. brucei, their mechanistic roles and the functional interplay among them remain poorly understood.

In this report, we investigated the requirement of the structural motifs in CIF3 for cytokinesis initiation, identified some interacting partners of CIF3, and studied the functional interplay between CIF3 and its interacting partner proteins. The results revealed distinct roles of the structural motifs of CIF3 in mediating the interaction with CIF3-interacting proteins and the recruitment of CIF3-interacting proteins to the initiation site of cytokinesis and demonstrated that CIF3-interacting proteins played essential roles in maintaining CIF3 at the cytokinesis initiation site. Together, these findings uncovered crucial roles of CIF3 in cytokinesis by cooperating with multiple cytokinesis regulators to promote cytokinesis initiation in T. brucei.

## RESULTS

### Determination of CIF3 structural motifs required for cytokinesis initiation.

**FIG 1:**
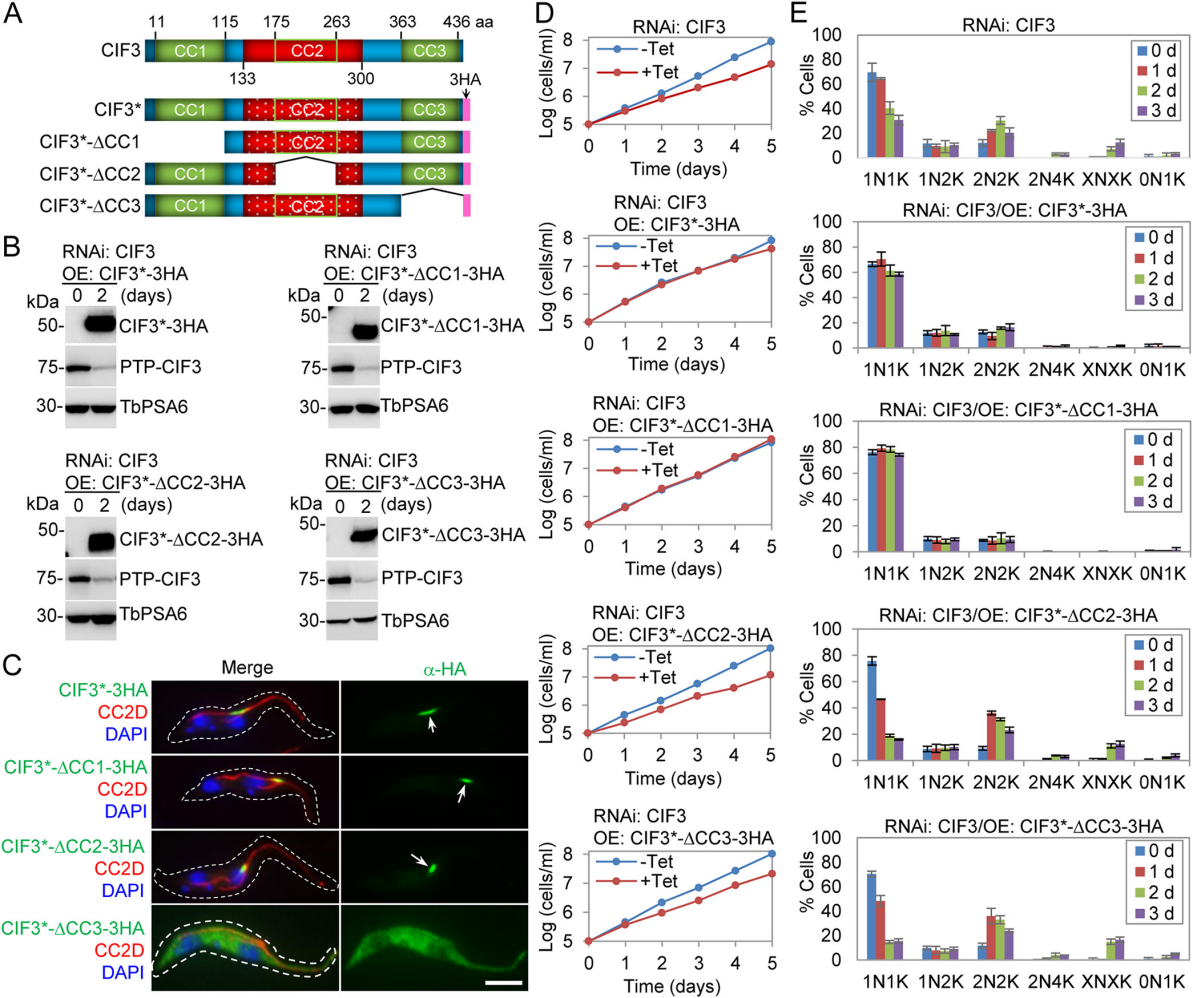
Identification of CIF3 structural motifs required for cytokinesis initiation. (A) Schematic illustration of CIF3 structural motifs and the full length and coiled-coil (CC) motif deletion mutants of recoded CIF3 (CIF3*) used for CIF3 RNAi complementation. The region marked in red (aa 133 to 300) corresponds to the DNA sequence targeted by CIF3 RNAi, whereas the region marked in patterned red represents the recoded sequence resistant to RNAi. (B) Western blotting to monitor the knockdown of CIF3 and ectopic expression of CIF3* and its CC deletion mutants. Endogenous CIF3 was tagged with an N-terminal PTP epitope and detected by anti-protein A antibody, and ectopic CIF3* and mutants were tagged with a C-terminal triple-HA epitope and detected by anti-HA antibody. TbPSA6 was detected by anti-TbPSA6 antibody and served as a loading control. (C) Subcellular localization of ectopically expressed CIF3* and its CC deletion mutants. The expression of CIF3* and its CC deletion mutants was induced with tetracycline (Tet) for 24 h. Cells were coimmunostained with FITC-conjugated anti-HA antibody and anti-CC2D antibody, which labels the FAZ filament, and counterstained with DAPI to label DNA. Arrows indicate CIF3* and its CC deletion mutants at the new FAZ tip. Bar, 5 μm. (D) Effect of CC motif deletion on cell proliferation. Shown are the growth curves of CIF3 RNAi and CIF3 RNAi complementation cell lines expressing CIF3* or its CC deletion mutants (CIF3*-ΔCC1, CIF3*-ΔCC2, and CIF3*-ΔCC3) before and after tetracycline induction for 5 days. (E) Effect of CC motif deletion on cell cycle progression. Shown are the tabulations of cells with different numbers of nuclei (N) and kinetoplasts (K) before and after tetracycline induction for 3 days. Two hundred cells from each time point were counted, and error bars indicate standard deviations (SD) from three independent experiments. OE, overexpression.

The essential cytokinesis regulator CIF3 contains three coiled-coil (CC) motifs, predicted by the DeepCoil and COILS algorithms, and no other conserved domains, as analyzed by hidden Markov models (Fig. 1A). It is unclear whether any of the three CC motifs are required for CIF3 function. To test the potential function of these CC motifs, we carried out genetic complementation of CIF3 deficiency by the ectopic expression of wild-type and CC deletion mutant CIF3 proteins in trypanosome cells. To this end, a CIF3 RNA interference (RNAi) cell line, which targeted CIF3 against the sequence encoding amino acids (aa) 134 to 300 of CIF3 (Fig. 1A), was generated (21). Subsequently, the endogenous CIF3 was tagged with an N-terminal PTP (Protein C-TEV-Protein A) epitope from one of its endogenous loci for monitoring the efficiency of CIF3 RNAi (21). Finally, the full length or CC deletion mutants of a recoded CIF3, designated CIF3*, in which each of the codons of amino acids 134 to 300 was replaced with a synonymous codon (Fig. 1A), was expressed from an ectopic locus in the CIF3 RNAi cell line. The cell lines thus generated are referred to here as CIF3 RNAi complementation cell lines. The ectopic expression of triple-hemagglutinin (3HA)-tagged CIF3* and its CC deletion mutants (CIF3*-ΔCC1, CIF3*-ΔCC2, and CIF3*-ΔCC3) and the knockdown of endogenous PTP-tagged CIF3 were confirmed by Western blotting with anti-HA antibody and anti-protein A (anti-ProtA) antibody, respectively (Fig. 1B). Coimmunofluorescence microscopy, with the FAZ protein CC2D (7) as the FAZ marker, showed that CIF3*, CIF3*-ΔCC1, and CIF3*-ΔCC2 localized to the distal tip of the new FAZ (Fig. 1C), whereas CIF3*-ΔCC3 was not localized to the new FAZ tip but instead was detected in the cytosol (Fig. 1C), indicating that the deletion of CC3, but not CC1 and CC2, impaired CIF3 localization. We next investigated the requirement of the three CC domains for CIF3 function in cell proliferation. Using these CIF3 RNAi complementation cell lines, we found that the expression of CIF3* and CIF3*-ΔCC1, but not CIF3*-ΔCC2 or CIF3*-ΔCC3, was able to restore cell proliferation (Fig. 1D), demonstrating that CC2 and CC3 are essential for CIF3 functions. The knockdown of CIF3 caused defects in cytokinesis initiation, resulting in the accumulation of cells with two nuclei and two kinetoplasts (2N2K) and multinucleated cells with multiple kinetoplasts (XNXK [X > 2]) (Fig. 1E). The expression of CIF3* or CIF3*-ΔCC1 restored cell division, whereas the expression of CIF3*-ΔCC2 or CIF3*-ΔCC3 resulted in the accumulation of 2N2K and XNXK cells (Fig. 1E), demonstrating that CC2 and CC3 are each required for CIF3 function in cytokinesis.

### Identification of CIF3 structural motifs involved in the interaction with CIF1 and TbPLK.

**FIG 2:**
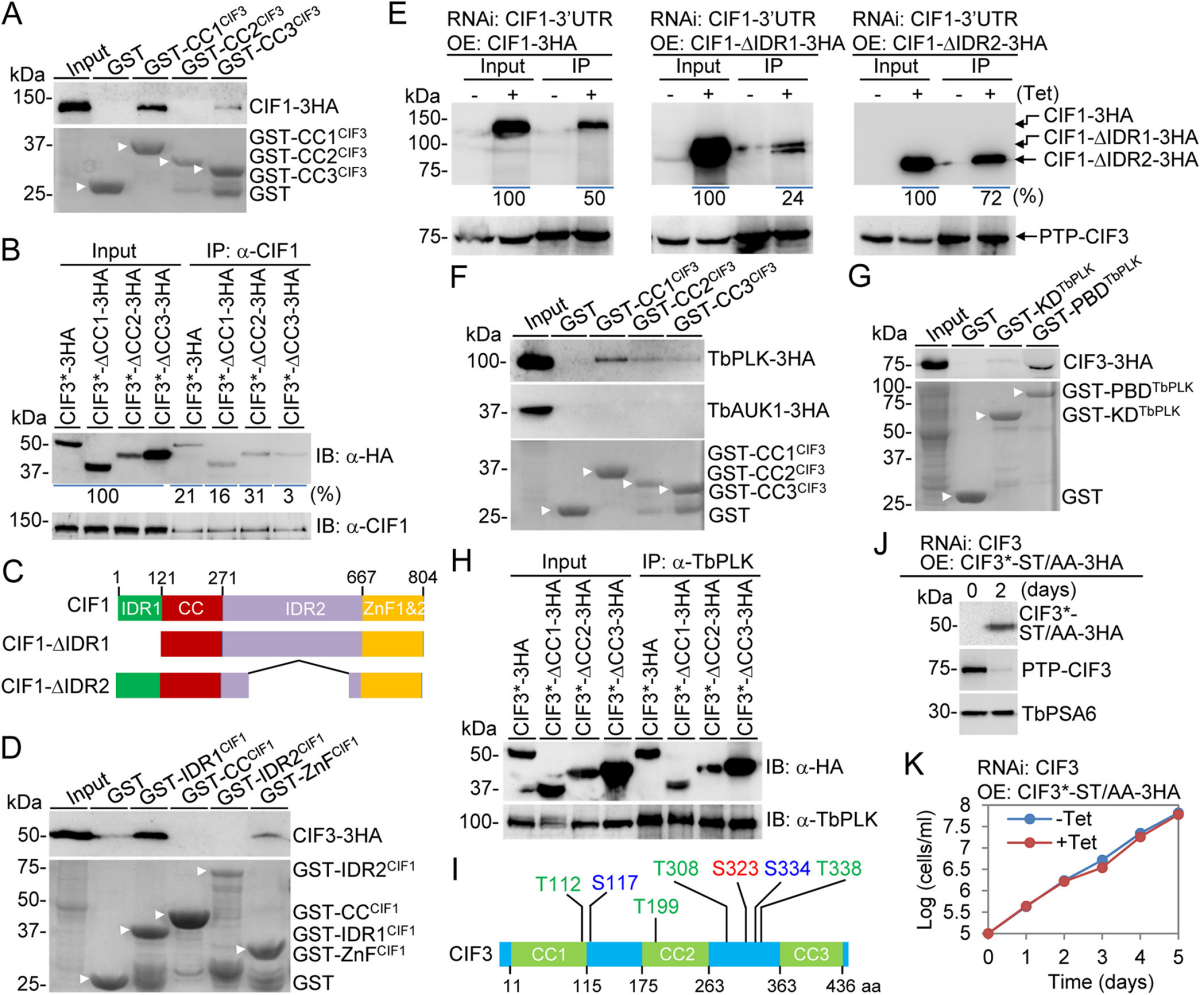
Determination of CIF3 structural motifs required for interaction with CIF1 and TbPLK. (A) GST pulldown experiments to test the CC motifs in CIF3 involved in interaction with CIF1. CIF1-3HA was detected by anti-HA antibody. White arrowheads indicate Coomassie blue-stained GST and GST-fused CC motifs of CIF3. (B) Coimmunoprecipitation to test the interaction of CIF3* and its CC deletion mutants with CIF1. CIF1 was immunoprecipitated (IP) and detected by immunoblotting (IB) with anti-CIF1 antibody, and 3HA-tagged CIF3* and CC deletion mutants were detected by immunoblotting with anti-HA antibody. The numbers under the anti-HA Western blot indicate the percentages of immunoprecipitated proteins of the input proteins (set as 100%). (C) Schematic drawing of CIF1 structural motifs and the deletion mutants of CIF1 used for coimmunoprecipitation. CC, coiled coil; IDR, intrinsically disordered region; ZnF, zinc finger. (D) GST pulldown experiments to test the CIF1 structural motifs involved in interaction with CIF3. CIF3-3HA was detected by anti-HA antibody. White arrowheads indicate Coomassie blue-stained GST and GST-fused structural motifs of CIF1. (E) Coimmunoprecipitation to test the interaction of CIF1, CIF1-ΔNTD, and CIF1-ΔIDR with CIF3. PTP-tagged CIF3 was immunoprecipitated by IgG beads and detected by anti-protein A antibody, and 3HA-tagged CIF1, CIF1-ΔNTD, and CIF1-ΔIDR were detected by anti-HA antibody. The numbers under the anti-HA Western blot indicate the percentages of immunoprecipitated proteins of the input proteins (set as 100%). (F) GST pulldown experiments to test the CIF3 structural motifs involved in interaction with TbPLK and TbAUK1. TbPLK-3HA and TbAUK1-3HA were each detected by anti-HA antibody. White arrowheads indicate Coomassie blue-stained GST and GST-fused CC motifs of CIF3. (G) GST pulldown experiments to test the TbPLK structural domains involved in interaction with CIF3. CIF3-3HA was detected by anti-HA antibody. White arrowheads indicate Coomassie blue-stained GST and the GST-fused kinase domain (KD) and Polo-box domain (PBD) of TbPLK. (H) Coimmunoprecipitation to test the interaction of CIF3* and its CC deletion mutants with TbPLK. TbPLK was immunoprecipitated and detected by immunoblotting with anti-TbPLK antibody, and 3HA-tagged CIF3* and CC deletion mutants were detected by immunoblotting with anti-HA antibody. (I) List of TbPLK phosphosites on CIF3 identified by an *in vitro* kinase assay and the *in vivo* phosphosites on CIF3 identified by phosphoproteomics in a previous study (28). The *in vitro* kinase assay was performed using recombinant GST-CIF3 purified from E. coli and TbPLK immunoprecipitated from trypanosome cells. *In vitro* TbPLK phosphosites were identified by mass spectrometry. Green, *in vitro* TbPLK phosphosite; blue, *in vivo* phosphosite; red, *in vitro* TbPLK phosphosite and *in vivo* phosphosite. (J) Western blotting to examine the knockdown of CIF3 by RNAi and ectopic expression of a 3HA-tagged phosphodeficient mutant (ST/AA) of CIF3*. Endogenously PTP-tagged CIF3 was detected by anti-protein A antibody, and CIF3*-ST/AA-3HA was detected by anti-HA antibody. TbPSA6 served as a loading control. (K) Effect of CIF3 phosphodeficient mutation on cell proliferation. Shown are the growth curves of the CIF3 RNAi cell line expressing CIF3*-ST/AA-3HA incubated without (−Tet) or with (+Tet) tetracycline for 5 days. OE, overexpression.

We previously demonstrated that CIF3 interacted with CIF1 *in vivo* in trypanosomes (21). To test which CC motifs in CIF3 mediate the interaction with CIF1, we performed an *in vitro* pulldown experiment using glutathione *S*-transferase (GST)-fused CC motifs and found that CC1 and CC3, but not CC2, were able to pull down CIF1 (Fig. 2A), indicating that CC1 and CC3 mediate the interaction with CIF1. Furthermore, we performed coimmunoprecipitation (co-IP) between CIF1 and the three CC deletion mutants of CIF3, and the results showed that each of the three CC deletion mutants was precipitated by CIF1, albeit the relative amount (∼3%) of precipitated CIF3-ΔCC3 was smaller than those of full-length CIF3 (∼21%) and the other two CC deletion mutants (∼16% and ∼31%) (Fig. 2B). This result suggests that both CC1 and CC3 of CIF3 contribute to the interaction with CIF1 *in vivo* in trypanosomes, and the deletion of CC3 in CIF3 may weaken the interaction of CIF3 with CIF1.

Conversely, we examined the structural motifs in CIF1 required for the interaction with CIF3. CIF1 contains a CC motif; two zinc finger (ZnF) motifs (ZnF1 and -2), which mediate the interaction with multiple cytokinesis regulators (18, 26); and two intrinsically disordered sequences located at the N terminus and between the CC and the ZnF motifs (named IDR1 and IDR2, respectively, here) (Fig. 2C). *In vitro* GST pulldown experiments showed that the IDR1 and the ZnF motifs, but not the CC and the IDR2 motifs, were able to precipitate CIF3 (Fig. 2D), indicating that IDR1 and the ZnF motifs mediate interactions with CIF3, in agreement with our previous report that one of the two ZnF motifs is required for the interaction with CIF3 *in vivo* in trypanosomes (21). To test whether IDR1 and IDR2 are required for the interaction with CIF3 *in vivo* in trypanosomes, we generated CIF1 3′-untranslated region (CIF1-3′UTR) RNAi complementation cell lines that express 3HA-tagged wild-type CIF1 or IDR1 deletion or IDR2 deletion mutants of CIF1 from an ectopic locus and PTP-tagged CIF3 from the endogenous locus. Immunoprecipitation of CIF3 was able to pull down CIF1, CIF1-ΔIDR1, and CIF1-ΔIDR2, albeit the relative amount (∼24%) of precipitated CIF1-ΔIDR1 was much smaller than those of CIF1 (∼50%) and CIF1-ΔIDR2 (∼72%) (Fig. 2E), suggesting that the interaction between CIF3 and CIF1 may be weakened by the deletion of IDR1 in CIF1.

We previously reported that TbPLK depletion impaired CIF3 localization to the new FAZ tip, and CIF3 depletion disrupted TbAUK1 localization to the new FAZ tip (21). We tested whether CIF3 interacts with TbPLK and TbAUK1 by GST pulldown experiments. The results showed that all three CC motifs were able to pull down TbPLK but not TbAUK1 (Fig. 2F). Conversely, the Polo-box domain (PBD), but not the kinase domain (KD), of TbPLK was able to pull down CIF3 (Fig. 2G). Furthermore, we carried out coimmunoprecipitations to test the interaction of TbPLK with CIF3 and its CC deletion mutants, and the results showed that immunoprecipitation of TbPLK was able to pull down CIF3 and all three CC deletion mutants of CIF3 (Fig. 2H), confirming that TbPLK and CIF3 interact *in vivo* in trypanosomes and demonstrating that the presence of any two of the three CC motifs is sufficient to maintain the TbPLK-CIF3 interaction in cells.

The Polo-like kinases from various organisms are known to bind to their substrates through the PBD (27), and CIF3 also binds to the PBD of TbPLK (Fig. 2G), suggesting that CIF3 might be a substrate of TbPLK. To test this possibility, we performed an *in vitro* kinase assay using recombinant CIF3 purified from bacteria and TbPLK immunoprecipitated from trypanosomes and identified five TbPLK phosphosites on CIF3, Thr112, Thr199, Thr308, Ser323, and Thr338 (Fig. 2I), among which Ser323 is one of the three *in vivo* phosphosites in CIF3 identified by phosphoproteomics (28). To test whether TbPLK phosphorylation of CIF3 is required for CIF3 function, we carried out complementation experiments by expressing CIF3* protein bearing point mutations of all five TbPLK phosphosites (CIF3*-ST/AA) in the CIF3 RNAi cell line. The tetracycline-induced knockdown of CIF3, which was endogenously tagged with a PTP epitope, and the ectopic expression of 3HA-tagged CIF3*-ST/AA were confirmed by Western blotting (Fig. 2J). The expression of CIF3*-ST/AA in CIF3-depleted cells was able to restore cell proliferation (Fig. 2K), demonstrating that TbPLK-mediated phosphorylation of these five sites is not essential for CIF3 function. Although we cannot rule out the possibility that some of the identified phosphosites might be phosphorylated by a contaminating protein kinase from either the T. brucei lysate or Escherichia coli, the results still demonstrate that phosphorylation of CIF3 at these sites was not essential for CIF3 function.

### Coiled-coil motif 3 in CIF3 is required for localizing CIF1 and TbPLK to the new FAZ tip.

**FIG 3:**
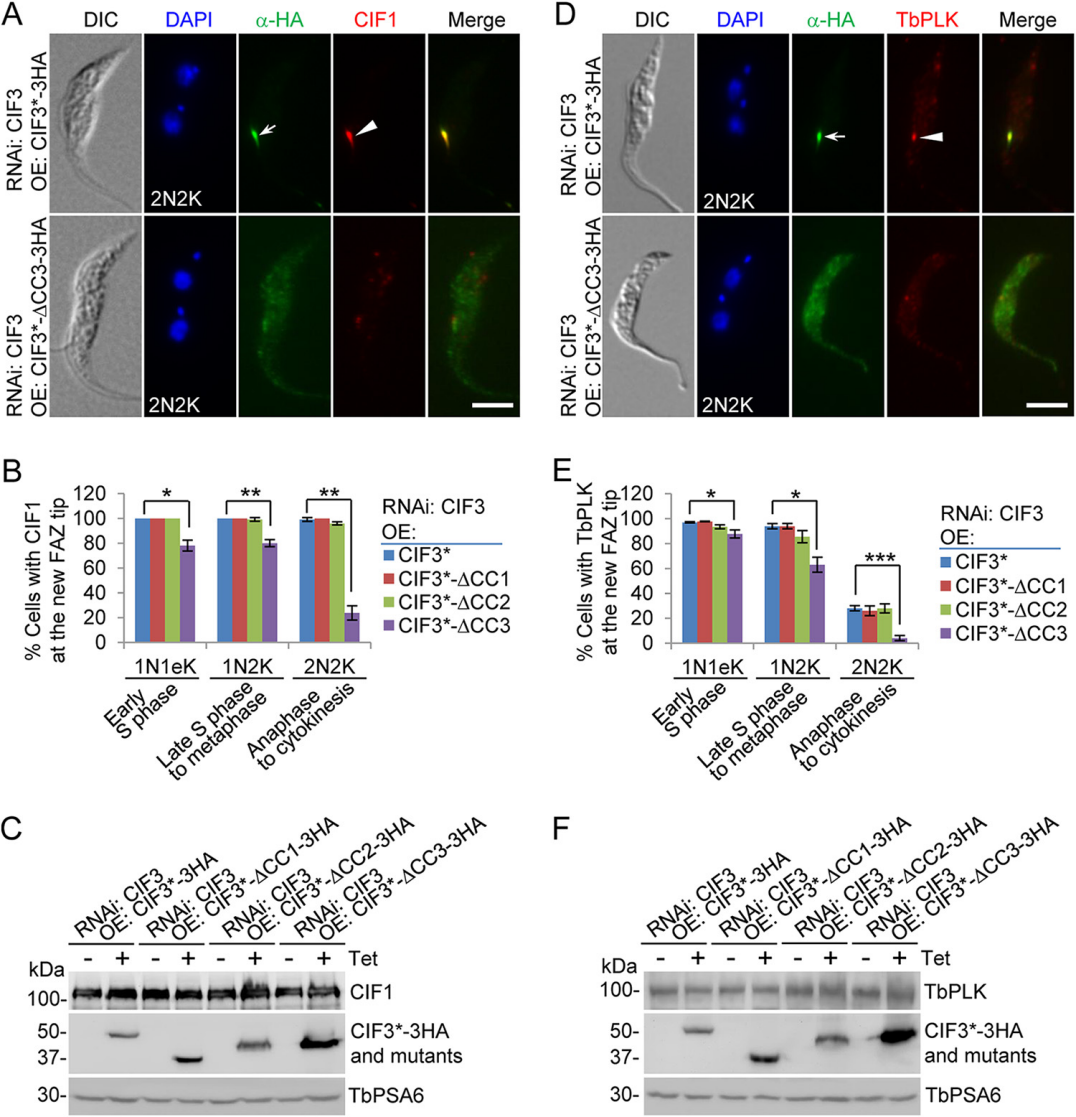
Coiled-coil motif 3 of CIF3 is required for the localization of CIF1 and TbPLK to the new FAZ tip. (A) CIF1 localization in CIF3 RNAi-induced cells expressing CIF3*-3HA or CIF3*-ΔCC3-3HA from an ectopic locus. Cells were induced with tetracycline for 48 h and coimmunostained with anti-HA antibody and anti-CIF1 antibody. The white arrow and arrowhead indicate CIF3*-3HA and CIF1 signals at the new FAZ tip, respectively. Bar, 5 μm. (B) Quantitation of cells with CIF1 localized at the new FAZ tip in CIF3 RNAi cells expressing CIF3*-3HA, CIF3*-ΔCC1-3HA, CIF3*-ΔCC2-3HA, or CIF3*-ΔCC3-3HA after 48 h of tetracycline induction. One hundred cells for each cell type (1N1eK, 1N2K, and 2N2K [eK indicates an elongated kinetoplast]) were counted, and error bars indicate SD from three independent experiments. *, *P* < 0.05; **, *P* < 0.01. (C) Western blotting to detect CIF1 protein levels in CIF3 RNAi cell lines expressing CIF3*-3HA, CIF3*-ΔCC1-3HA, CIF3*-ΔCC2-3HA, or CIF3*-ΔCC3-3HA without (−) and with (+) tetracycline (Tet) induction for 48 h. CIF1 was detected by anti-CIF1 antibody, and 3HA-tagged CIF3* and its CC deletion mutants were detected by anti-HA antibody. TbPSA6 served as a loading control. (D) TbPLK localization in CIF3 RNAi-induced cells expressing CIF3*-3HA or CIF3*-ΔCC3-3HA from an ectopic locus. Cells were induced with tetracycline for 48 h and coimmunostained with anti-HA antibody and anti-TbPLK antibody. The white arrow and arrowhead indicate CIF3*-3HA and TbPLK signals at the new FAZ tip, respectively. Bar, 5 μm. (E) Quantitation of cells with TbPLK localized at the new FAZ tip in CIF3 RNAi cells expressing CIF3*-3HA, CIF3*-ΔCC1-3HA, CIF3*-ΔCC2-3HA, or CIF3*-ΔCC3-3HA after 48 h of tetracycline induction. One hundred cells for each cell type (1N1eK, 1N2K, and 2N2K) were counted, and error bars indicate SD from three independent experiments. *, *P* < 0.05; ***, *P* < 0.001. (F) Western blotting to detect TbPLK protein levels in CIF3 RNAi cell lines expressing CIF3*-3HA, CIF3*-ΔCC1-3HA, CIF3*-ΔCC2-3HA, or CIF3*-ΔCC3-3HA without and with tetracycline induction for 48 h. TbPLK was detected by anti-TbPLK antibody, and 3HA-tagged CIF3* and its CC deletion mutants were detected by anti-HA antibody. TbPSA6 served as a loading control. DIC, differential interference contrast; OE, overexpression.

We investigated the requirement of CIF3 structural motifs for the localization of CIF1 and TbPLK to the new FAZ tip using the three CIF3 RNAi complementation cell lines. Immunofluorescence microscopy using anti-CIF1 antibody showed that CIF1 was detected at the new FAZ tip in almost all cells for the CIF3 RNAi-induced cells expressing CIF3*-ΔCC1 or CIF3*-ΔCC2 (Fig. 3A and B). However, in the CIF3 RNAi-induced cells expressing CIF3*-ΔCC3, the percentages of cells with a detectable CIF1 signal at the new FAZ tip were reduced by ∼20% during early S phase to metaphase and by ∼76% during anaphase to cytokinesis (Fig. 3A and B). Western blotting showed that the CIF1 protein level was not affected in the CIF3 RNAi cell line expressing CIF3*-ΔCC3 before and after tetracycline induction (Fig. 3C). Similarly, immunofluorescence microscopy using anti-TbPLK antibody showed that TbPLK localization to the new FAZ tip was not affected in the CIF3 RNAi-induced cells expressing CIF3*-ΔCC1 or CIF3*-ΔCC2 (Fig. 3D and E). In contrast, in the CIF3 RNAi-induced cells expressing CIF3*-ΔCC3, the percentages of cells with a detectable TbPLK signal at the new FAZ tip were reduced from ∼97% to ∼88% among early S-phase cells, from ∼94% to ∼63% among late S phase-to-metaphase cells, and from ∼28% to ∼4% among anaphase-to-cytokinesis cells (Fig. 3D and E). The lower percentage of TbPLK-positive cells in the control background is because TbPLK is expressed before early anaphase but not in late anaphase until cytokinesis (29, 30). Western blotting showed that the protein level of TbPLK was not changed in any of these CIF3 RNAi complementation cell lines before and after the induction of CIF3 RNAi and the expression of full-length CIF3* and CC deletion mutants of CIF3* (Fig. 3F). These results suggest that the deletion of CC3 in CIF3 disrupted the localization of CIF1 and TbPLK to the new FAZ tip, causing the distribution of the two proteins to the cytosol.

### The cytokinesis regulators CIF4, FPRC, and KAT80 are CIF3-interacting partners.

**FIG 4:**
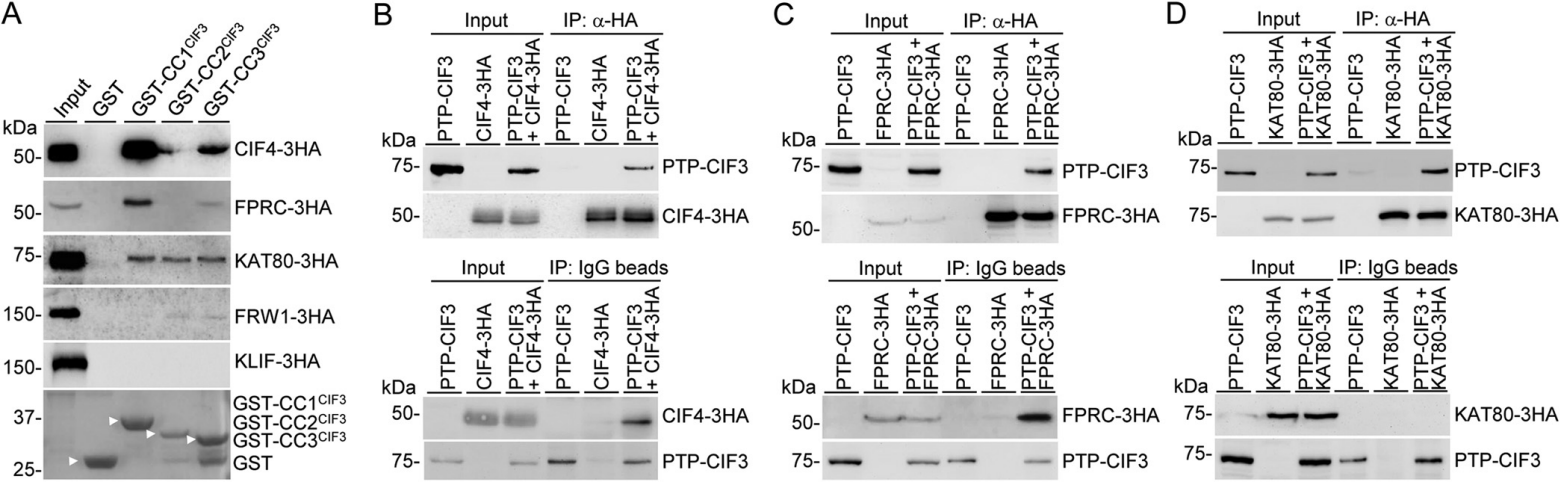
Identification of CIF3-interacting cytokinesis regulatory proteins by GST pulldown and coimmunoprecipitation. (A) GST pulldown experiments to test the interaction of CIF3 with various cytokinesis regulators. GST-fused CC motifs of CIF3 (stained with Coomassie blue and indicated by white arrowheads) were used as bait, and 3HA-tagged CIF4, FPRC, KAT80, FRW1, and KLIF were used as prey and were detected by anti-HA antibody. (B) Coimmunoprecipitation of CIF3 and CIF4 in trypanosomes. Cells coexpressing PTP-CIF3 and CIF4-3HA were used for immunoprecipitation (IP) with anti-HA beads or IgG beads to pull down CIF4-3HA or PTP-CIF3, followed by immunoblotting with anti-protein A antibody and anti-HA antibody to detect PTP-CIF3 and CIF4-3HA, respectively. (C) Coimmunoprecipitation of CIF3 and FPRC in trypanosomes. Cells coexpressing PTP-CIF3 and FPRC-3HA were used for IP, as described above for panel B. (D) Coimmunoprecipitation of CIF3 and KAT80 in trypanosomes. Cells coexpressing PTP-CIF3 and KAT80-3HA were used for IP, as described above for panel B.

Cytokinesis initiation in trypanosomes is regulated by a cohort of trypanosome-specific proteins located at the new FAZ tip (17–23), and CIF1 appears to interact with multiple cytokinesis regulators *in vivo* in trypanosomes (18, 20, 21, 23). We asked whether CIF3 also interacts with multiple cytokinesis regulators in addition to CIF1 and TbPLK (Fig. 2). *In vitro* pulldown experiments using GST-fused CC motifs of CIF3 identified CIF4, FPRC, and KAT80, but not KLIF, as CIF3-interacting proteins, with the CC1 and CC3 motifs mediating the interaction with CIF4 and FPRC and all three CC motifs mediating the interaction with KAT80 (Fig. 4A). A very small amount of FRW1 protein was pulled down by CC2 and CC3 of CIF3 (Fig. 4A), which could be due to a nonspecific association, and hence, it was not considered an interacting partner of CIF3.

To test whether CIF3 interacts with CIF4 *in vivo* in trypanosomes, we performed coimmunoprecipitation using cells coexpressing PTP-CIF3 and CIF4-3HA from their respective endogenous loci. Immunoprecipitation of CIF4-3HA with anti-HA antibody was able to pull down PTP-CIF3 from the trypanosome cell lysate, and reciprocally, immunoprecipitation of PTP-CIF3 with IgG beads was able to pull down CIF4-3HA from the cell lysate (Fig. 4B), confirming that CIF3 and CIF4 form a complex *in vivo* in trypanosomes. To test whether CIF3 and FPRC interact *in vivo* in trypanosomes, we performed similar coimmunoprecipitation experiments using cells coexpressing PTP-CIF3 and FPRC-3HA from their respective endogenous loci. Immunoprecipitation of FPRC-3HA with anti-HA antibody was able to pull down PTP-CIF3 from the trypanosome cell lysate, and reciprocally, immunoprecipitation of PTP-CIF3 with IgG beads was able to pull down FPRC-3HA from the cell lysate (Fig. 4C), confirming that CIF3 and FPRC interact *in vivo* in trypanosomes. Finally, to test whether CIF3 and KAT80 also interact *in vivo* in trypanosomes, we performed coimmunoprecipitation experiments using cells coexpressing PTP-CIF3 and KAT80-3HA from their respective endogenous loci to test whether they form a complex in trypanosomes. Immunoprecipitation of KAT80-3HA with anti-HA antibody was able to pull down PTP-CIF3 (Fig. 4D). However, the reciprocal immunoprecipitation with IgG beads to pull down PTP-CIF3 was unable to coprecipitate KAT80-3HA (Fig. 4D). One possible reason for this could be that the IgG bead binding of PTP-CIF3 somehow interfered with the KAT80-binding site on CIF3, which disrupted their interaction. Nonetheless, the results from the anti-HA coimmunoprecipitation experiments suggest that CIF3 and KAT80 interact *in vivo* in trypanosomes. Taken together, the GST pulldown and coimmunoprecipitation experiments (Fig. 2 and 4) demonstrated that CIF3 interacts with the cytokinesis regulators TbPLK, CIF1, CIF4, FPRC, and KAT80 but not the cytokinesis regulators TbAUK1, FRW1, and KLIF, indicating that CIF3 may cooperate with multiple cytokinesis regulators to promote cytokinesis initiation.

### CIF3 is required for CIF4 localization to the new FAZ tip, whereas CIF4 maintains CIF3 at the new FAZ tip.

**FIG 5:**
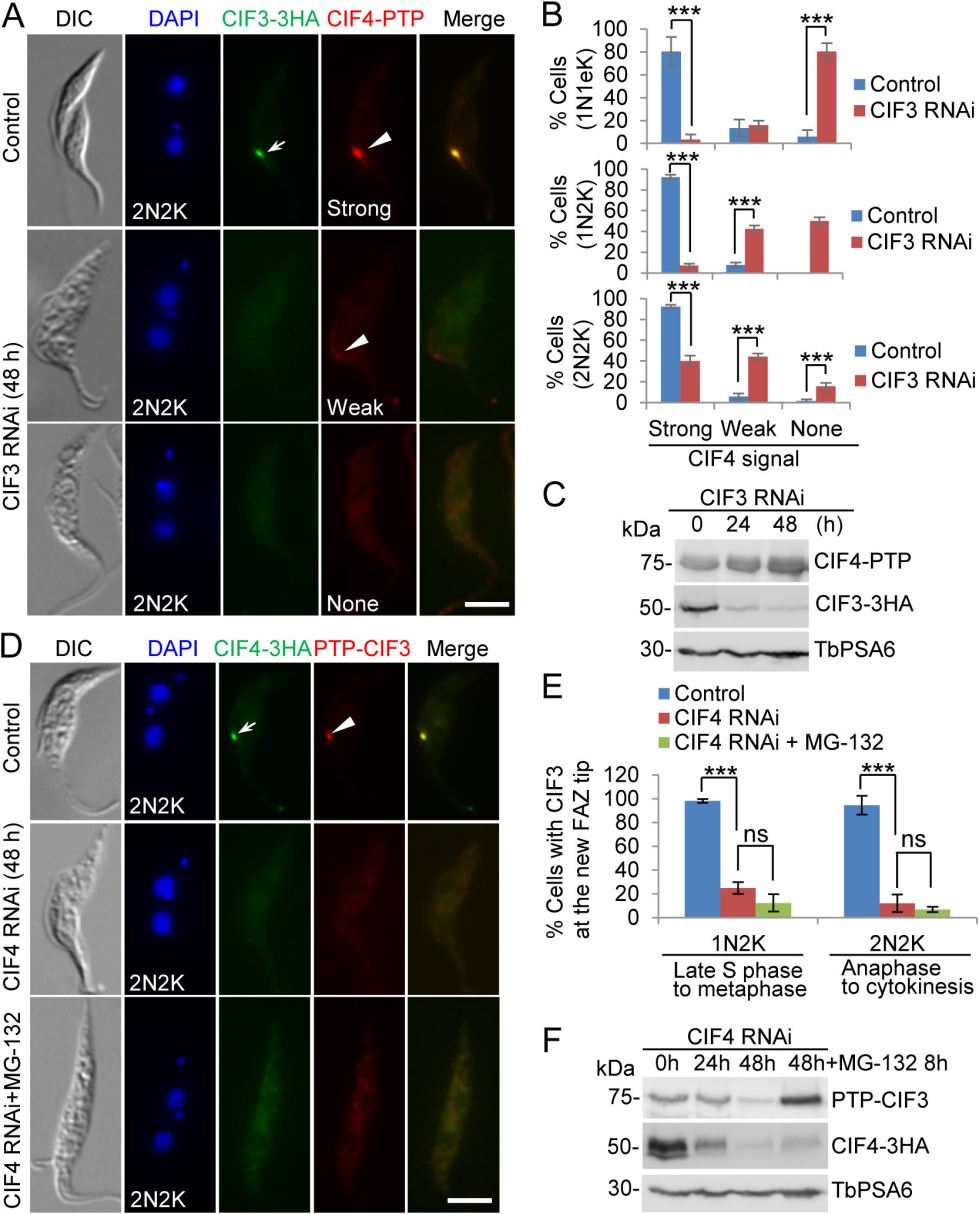
CIF3 is required for CIF4 localization to the new FAZ tip, and CIF4 maintains CIF3 stability. (A) Effect of CIF3 RNAi on CIF4 localization. Noninduced control and CIF3 RNAi-induced cells (48 h) were coimmunostained with anti-HA antibody and anti-protein A antibody to detect CIF3-3HA and CIF4-PTP, respectively. The white arrow and arrowheads indicate CIF3-3HA and CIF4-PTP signals at the new FAZ tip, respectively. Bar, 5 μm. (B) Quantitation of cells with CIF4 signals of various intensities at the new FAZ tip in noninduced control and CIF3 RNAi-induced cells. One hundred cells of each cell type (1N1eK, 1N2K, and 2N2K) for each cell line were counted, and error bars indicate SD from three independent experimental replicates. ***, *P* < 0.001. (C) Western blotting to examine the effect of CIF3 RNAi on CIF4 protein stability. CIF4 and CIF3 were endogenously tagged with PTP and triple HA and detected by anti-protein A antibody and anti-HA antibody, respectively. TbPSA6 served as a loading control. (D) Effect of CIF4 RNAi on CIF3 localization. Noninduced control cells, CIF4 RNAi-induced cells (48 h), and CIF4 RNAi-induced cells (48 h) treated with MG-132 (8 h) were coimmunostained with anti-HA antibody and anti-protein A antibody to detect CIF4-3HA and PTP-CIF3, respectively. The white arrow and arrowhead indicate CIF4-3HA and PTP-CIF3 signals at the new FAZ tip, respectively. Bar, 5 μm. (E) Quantitation of cells with CIF3 at the new FAZ tip in noninduced control cells, CIF4 RNAi-induced cells, and CIF4 RNAi-induced cells treated with MG-132. One hundred cells of each cell type (1N2K and 2N2K) for each cell line were counted, and error bars indicate SD from three independent experimental replicates. ***, *P* < 0.001; ns, no significance. (F) Western blotting to examine the effect of CIF4 RNAi on CIF3 protein stability. CIF3 and CIF4 were endogenously tagged with PTP and triple HA and detected by anti-protein A antibody and anti-HA antibody, respectively. TbPSA6 served as a loading control.

Given that CIF3 and CIF4 form a complex in trypanosome cells, we investigated the functional relationship between CIF3 and CIF4 by examining the effect of the subcellular localization and stability of one protein in the absence of its partner protein. Immunofluorescence microscopy showed that the knockdown of CIF3 disrupted the localization of CIF4 in all cell types from early S phase (1N1eK cells, where eK indicates an elongated kinetoplast) to telophase (2N2K cells) after RNAi induction for 48 h (Fig. 5A), causing reductions of 1N1eK cells with detectable CIF4 at the new FAZ tip by ∼77%, 1N2K cells with detectable CIF4 at the new FAZ tip by ∼85%, and 2N2K cells with detectable CIF4 at the new FAZ tip by ∼52% (Fig. 5B). Consequently, cells without a CIF4 signal at the new FAZ tip were increased by ∼75% for 1N1eK cells, ∼50% for 1N2K cells, and ∼14% for 2N2K cells, respectively (Fig. 5B). Moreover, cells with a weak CIF4 signal at the new FAZ tip were increased by ∼35% in 1N2K cells and ∼38% in 2N2K cells but were only slightly (∼3%) increased in 1N1eK cells (Fig. 5B). Western blotting showed that the protein level of CIF4 was not affected in CIF3 RNAi cells (Fig. 5C), demonstrating that CIF3 depletion impaired CIF4 localization to the new FAZ tip.

Conversely, CIF4 knockdown decreased 1N2K cells with detectable CIF3 at the new FAZ tip by ∼73% and 2N2K cells with detectable CIF3 at the new FAZ tip by ∼82% after CIF4 RNAi induction for 48 h (Fig. 5D and E). Western blotting showed that the protein level of CIF3, which was endogenously tagged with PTP, was reduced after 24 h of CIF4 RNAi, and treatment of cells with the proteasome inhibitor MG-132 stabilized CIF3 protein (Fig. 5F). However, this treatment with MG-132 did not restore CIF3 localization to the new FAZ tip (Fig. 5D and E), indicating that CIF3 was spread out to the cytosol and then degraded in CIF4 RNAi-induced cells. These results suggest that the depletion of CIF4 disrupted the localization of CIF3 to the new FAZ tip, and CIF4 appeared to be required to maintain CIF3 stability.

**FIG 6:**
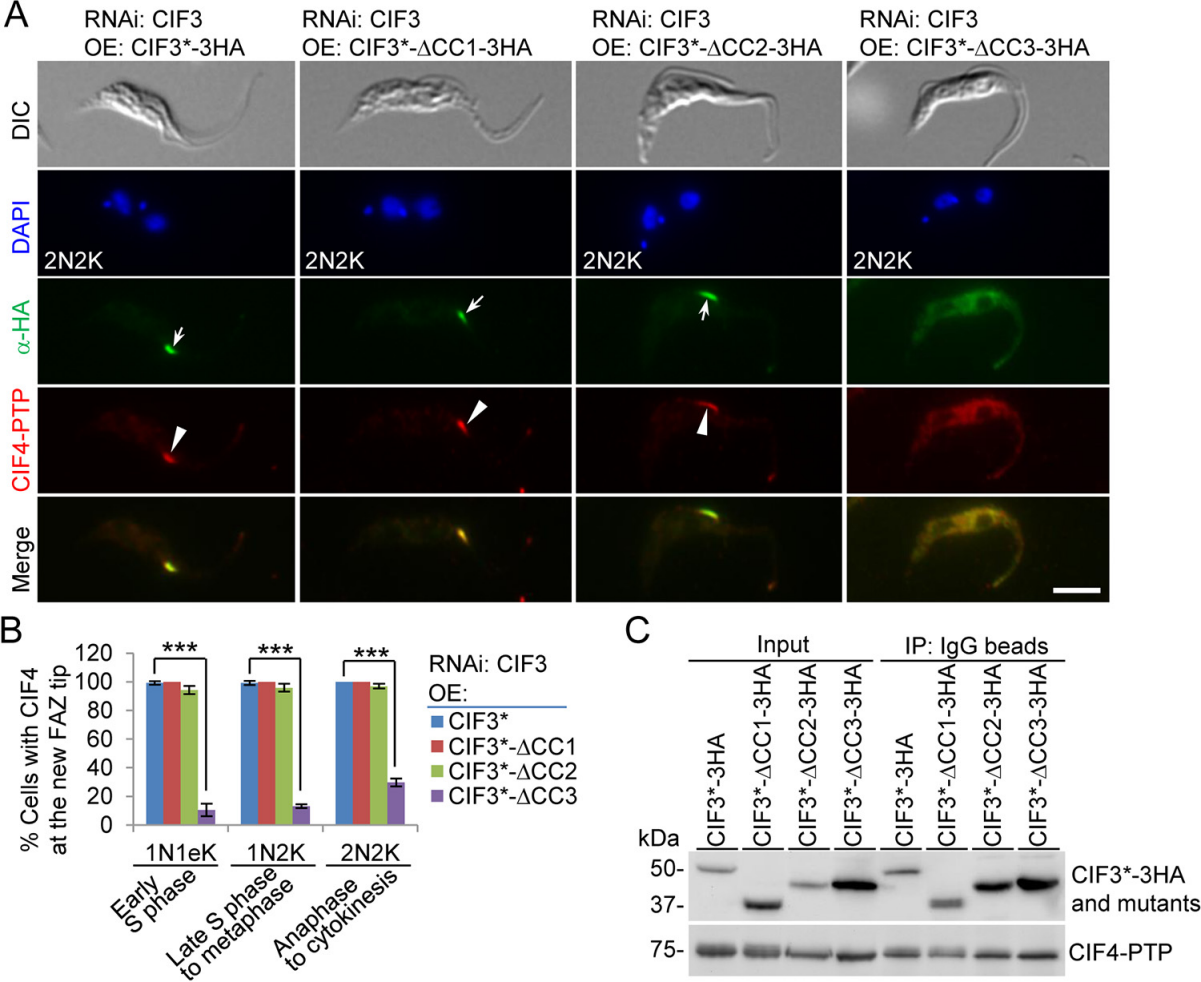
Determination of CIF3 structural motifs required for CIF4 localization to the new FAZ tip. (A) CIF4 localization in CIF3 RNAi-induced cells expressing CIF3*-3HA or its CC deletion mutants from an ectopic locus. CIF4 was endogenously tagged with a C-terminal PTP epitope in the CIF3 RNAi complementation cell lines. Cells were induced with tetracycline for 48 h and coimmunostained with anti-HA antibody and anti-protein A antibody. White arrows indicate 3HA-tagged CIF3* and its mutants at the new FAZ tip, whereas white arrowheads indicate CIF4-PTP at the new FAZ tip. Bar, 5 μm. (B) Quantitation of cells with CIF4 localized at the new FAZ tip in CIF3 RNAi cells expressing CIF3*-3HA, CIF3*-ΔCC1-3HA, CIF3*-ΔCC2-3HA, or CIF3*-ΔCC3-3HA after 48 h of tetracycline induction. One hundred cells for each cell type (1N1eK, 1N2K, and 2N2K) were counted, and error bars indicate SD from three independent experiments. ***, *P* < 0.001. (C) Coimmunoprecipitation to test the interaction of CIF3* and its CC deletion mutants with CIF4. CIF4 was endogenously tagged with PTP, immunoprecipitated with IgG beads, and detected by anti-protein A antibody, whereas 3HA-tagged CIF3* and its CC deletion mutants were detected by anti-HA antibody. OE, overexpression.

We next examined the requirement of the CC motifs in CIF3 for CIF4 localization using the CIF3 RNAi complementation cell lines that express PTP-tagged CIF4 from the endogenous locus. Immunofluorescence microscopy showed that in the CIF3 RNAi-induced cells expressing CIF3*-ΔCC3, but not CIF3*-ΔCC1 and CIF3*-ΔCC2, CIF4 was distributed into the cytosol (Fig. 6A). Quantitative analysis showed that cells with a detectable CIF4 signal at the new FAZ tip in the CIF3 RNAi cells expressing CIF3*-ΔCC3 were reduced by ∼89% in early S-phase cells (1N1eK cells), ∼86% in late S phase-to-metaphase cells (1N2K cells), and ∼70% in anaphase-to-cytokinesis cells (2N2K cells) (Fig. 6B). Immunoprecipitation with IgG beads to pull down CIF4-PTP was able to coprecipitate full-length CIF3 and its three CC deletion mutants (Fig. 6C), demonstrating that the deletion of each of the three CC motifs did not affect the interaction between CIF3 and CIF4. Given that both CC1 and CC3 of CIF3 can mediate the interaction with CIF4 (Fig. 4A), this suggests that the presence of either CC1 or CC3 is sufficient to maintain the interaction of CIF3 with CIF4 in trypanosome cells.

Together, the results obtained from the examination of CIF3 localization in CIF4 RNAi cells and CIF4 localization in CIF3 RNAi cells (Fig. 5) indicate that the presence of either protein in trypanosome cells is necessary for the localization of its interacting partner protein to the new FAZ tip and that the presence of CIF4 is additionally required for maintaining CIF3 stability but not vice versa. However, the results obtained from the examination of CIF4 localization in CIF3 RNAi complementation cell lines (Fig. 6) suggest that if both CIF3 and CIF4 proteins are present in trypanosome cells, CIF4 localization to the new FAZ tip depends on CIF3.

### CIF3 targets FPRC to the new FAZ tip, whereas FPRC maintains CIF3 at the new FAZ tip during late cell cycle stages.

**FIG 7:**
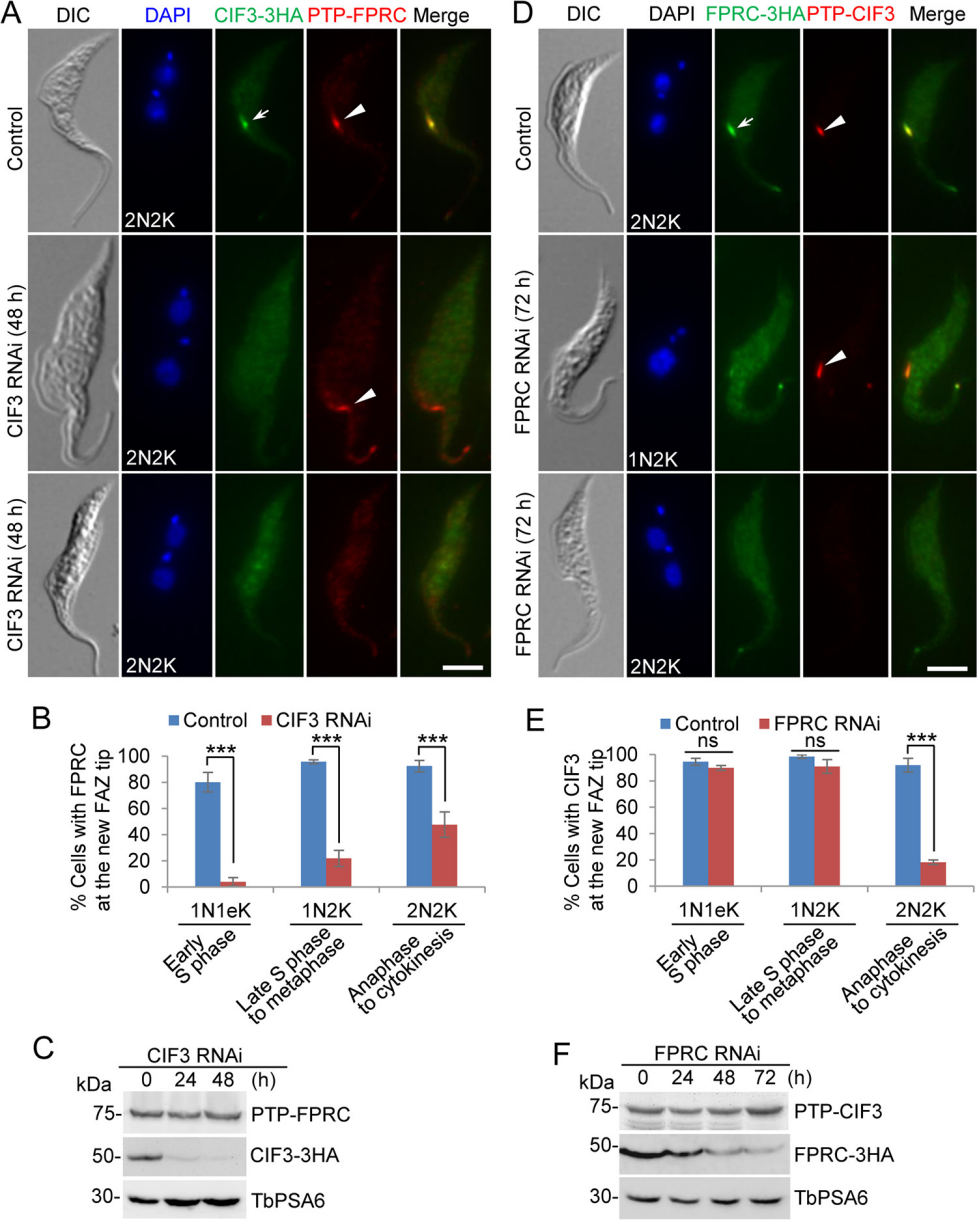
CIF3 and FPRC are interdependent for localization to the new FAZ tip. (A) FPRC localization in noninduced control and CIF3 RNAi-induced cells (48 h). The white arrow and arrowheads indicate CIF3-3HA and PTP-FPRC signals, respectively. Bar, 5 μm. (B) Quantitation of cells with FPRC at the new FAZ tip. One hundred cells of each cell type (1N1eK, 1N2K, and 2N2K) for each cell line were counted, and error bars indicate SD from three independent experimental replicates. ***, *P* < 0.001. (C) Western blotting to examine FPRC protein stability. FPRC and CIF3 were endogenously tagged with PTP and triple HA, respectively. TbPSA6 served as a loading control. (D) Effect of FPRC RNAi on CIF3 localization. Noninduced control and FPRC RNAi-induced cells (72 h) were used for coimmunostaining. The white arrow and arrowheads indicate FPRC-3HA and PTP-CIF3 signals, respectively. Bar, 5 μm. (E) Quantitation of cells with CIF3 signals at the new FAZ tip in noninduced control and FPRC RNAi-induced cells. One hundred cells of each cell type (1N1eK, 1N2K, and 2N2K) for each cell line were counted, and error bars indicate SD from three independent experimental replicates. ***, *P* < 0.001; ns, no significance. (F) Western blotting to examine CIF3 protein stability. CIF3 and FPRC were endogenously tagged with PTP and triple HA, respectively. TbPSA6 served as a loading control.

The interaction between CIF3 and FPRC *in vivo* in trypanosomes (Fig. 4) prompted us to examine the functional interplay between the two proteins. To this end, we first investigated the effect of CIF3 knockdown on the localization and stability of FPRC using the CIF3 RNAi cell line coexpressing PTP-tagged FPRC and triple-HA-tagged CIF3 from their respective endogenous loci. Immunofluorescence microscopic analysis of the noninduced and RNAi-induced cells showed that after CIF3 knockdown, cells with a detectable FPRC signal at the new FAZ tip were reduced by ∼76% for 1N1eK cells, ∼74% for 1N2K cells, and ∼45% for 2N2K cells (Fig. 7A and B). Western blotting showed that the protein level of FPRC was not affected in CIF3 RNAi cells (Fig. 7C), suggesting that FPRC protein was spread out into the cytosol after CIF3 RNAi induction in the majority of the RNAi cells. Therefore, CIF3 is required for FPRC localization to the new FAZ tip from early S phase onward until cytokinesis. It should be noted that the percentage of CIF3 RNAi cells with a detectable FPRC signal at the new FAZ tip was gradually increased during the cell cycle (Fig. 7B). This surprising result suggests that FPRC localization to the new FAZ tip becomes less dependent on CIF3 following cell cycle progression.

Conversely, we investigated the effect of FPRC knockdown on the localization and stability of CIF3 using the FPRC RNAi cell line coexpressing PTP-tagged CIF3 and triple-HA-tagged FPRC from their respective endogenous loci. Immunofluorescence microscopy showed that after FPRC was depleted by RNAi, cells with a detectable CIF3 signal at the new FAZ tip were not affected in 1N1eK cells and 1N2K cells but were significantly decreased in 2N2K cells (Fig. 7D and E). Western blotting showed that the protein level of CIF3 was not affected by FPRC RNAi (Fig. 7F). These results suggest that FPRC is not required for CIF3 localization to the new FAZ tip during early cell cycle stages from early S phase to metaphase, but it is necessary for CIF3 localization during late cell cycle stages from anaphase to cytokinesis. Given that CIF3 is recruited to the new FAZ tip as soon as the new FAZ is assembled at early S phase, these results imply that FPRC is not involved in recruiting and maintaining CIF3 from early S phase until metaphase, but it becomes essential for maintaining CIF3 at the new FAZ tip when the cell cycle progresses into anaphase until cytokinesis.

**FIG 8:**
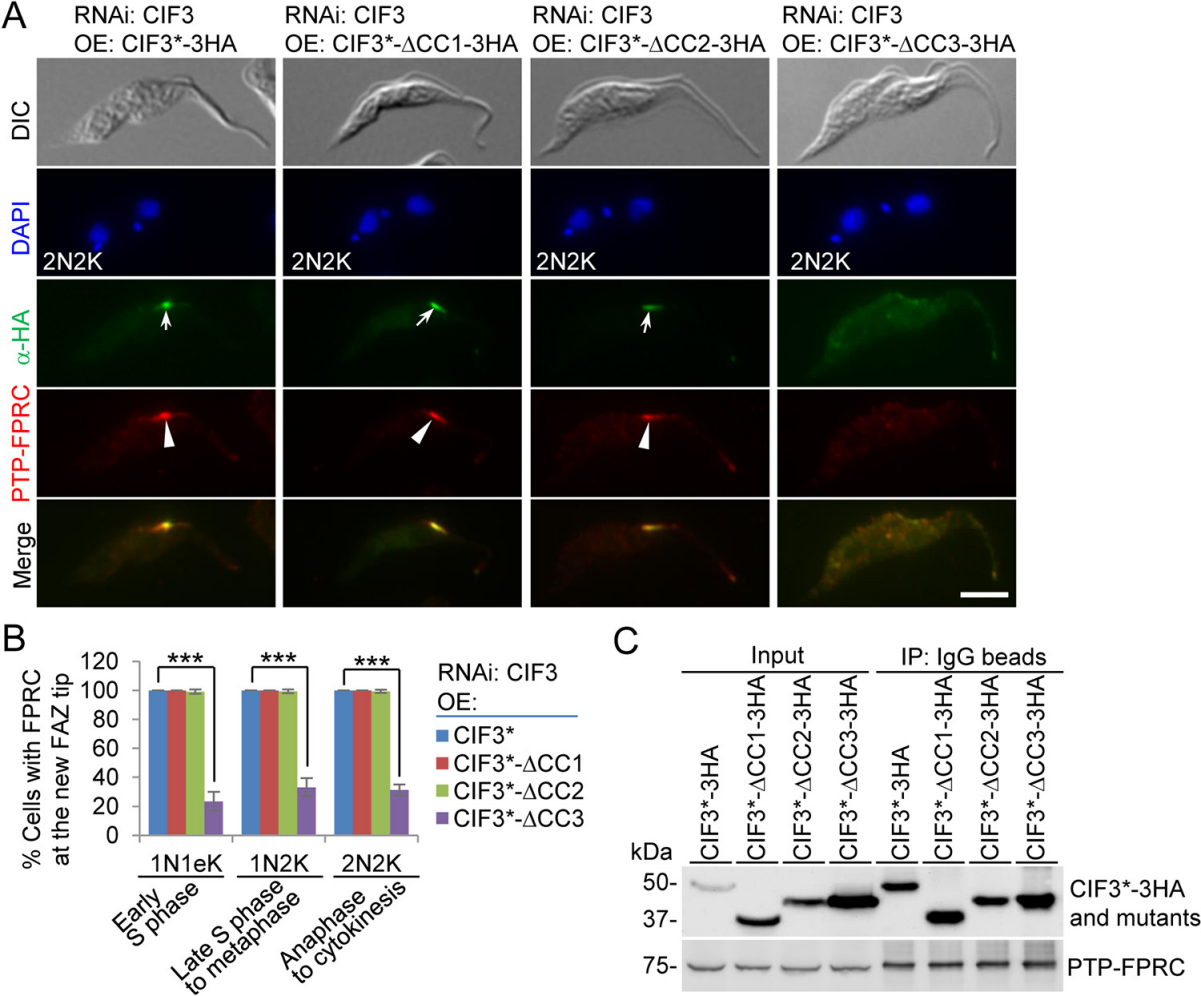
Determination of CIF3 structural motifs required for FPRC localization to the new FAZ tip. (A) FPRC localization in CIF3 RNAi-induced cells expressing CIF3*-3HA or its mutants from an ectopic locus. FPRC was endogenously tagged with an N-terminal PTP epitope in the CIF3 RNAi complementation cell lines. Cells were induced with tetracycline for 48 h and coimmunostained with anti-HA antibody and anti-protein A antibody. White arrows indicate 3HA-tagged CIF3* and its mutants at the new FAZ tip, whereas white arrowheads indicate PTP-FPRC at the new FAZ tip. Bar, 5 μm. (B) Quantitation of cells with FPRC localized at the new FAZ tip in CIF3 RNAi cells expressing CIF3*-3HA, CIF3*-ΔCC1-3HA, CIF3*-ΔCC2-3HA, or CIF3*-ΔCC3-3HA after 48 h of tetracycline induction. One hundred cells of each cell type (1N1eK, 1N2K, and 2N2K) were counted, and error bars indicate SD from three independent experiments. ***, *P* < 0.001. (C) Coimmunoprecipitation to test the interaction of CIF3* and its CC deletion mutants with FPRC. FPRC was endogenously tagged with PTP, immunoprecipitated with IgG beads, and detected by anti-protein A antibody, whereas 3HA-tagged CIF3* and its CC deletion mutants were detected by anti-HA antibody. OE, overexpression.

To further confirm the results obtained from the RNAi cell lines described above, we examined the effect of the deletion of the CIF3 coiled-coil motif on the localization of FPRC and the interaction between CIF3 and FPRC. To this end, FPRC was endogenously tagged with an N-terminal PTP epitope in the CIF3 RNAi complementation cell lines generated as described above (Fig. 1), and the resulting cell lines were used for immunofluorescence microscopy and coimmunoprecipitation. The deletion of CC1 or CC2 from CIF3 did not affect the localization of FPRC, but the deletion of CC3 from CIF3 impaired the localization of FPRC (Fig. 8A), causing reductions of cells with a detectable FPRC signal at the new FAZ tip by ∼77% for 1N1eK cells, ∼67% for 1N2K cells, and ∼69% for 2N2K cells (Fig. 8B). Immunoprecipitation of PTP-tagged FPRC with IgG beads was able to pull down 3HA-tagged CIF3* and all three CC deletion mutants of CIF3* (Fig. 8C), demonstrating that the deletion of any of the three CC motifs did not affect the interaction with FPRC. Altogether, these results suggest that FPRC localization to the new FAZ tip depends on its interaction with CIF3; thus, CIF3 targets FPRC to the new FAZ tip during S phase and maintains FPRC at the new FAZ tip thereafter.

### CIF3 targets KAT80 to the new FAZ tip, whereas KAT80 maintains CIF3 at the new FAZ tip at late cell cycle stages.

**FIG 9:**
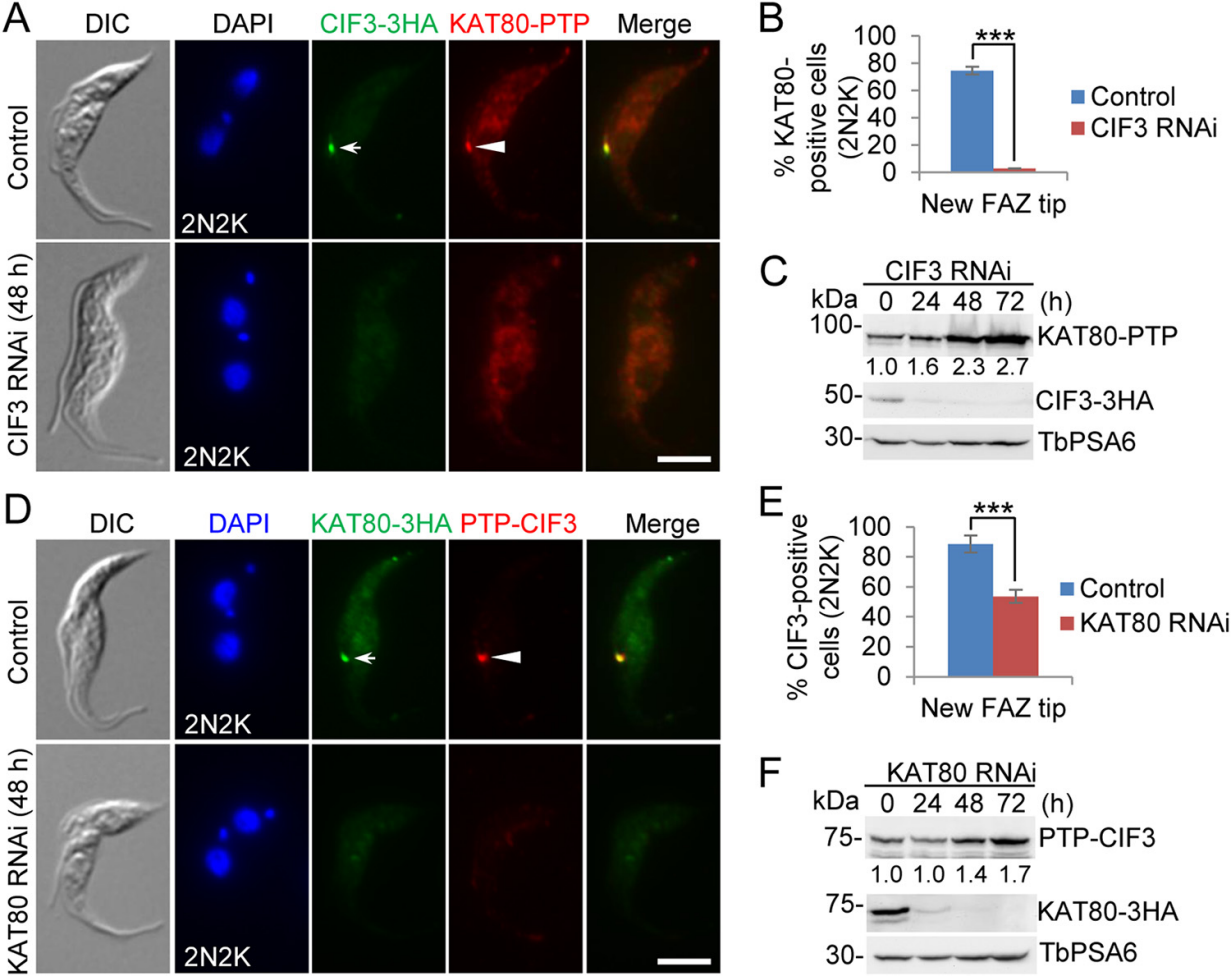
Effects of CIF3 RNAi on KAT80 localization and KAT80 RNAi on CIF3 localization. (A) Effect of CIF3 RNAi on KAT80 localization. Noninduced control and CIF3 RNAi-induced cells (48 h) were used for coimmunostaining. The white arrow and arrowhead indicate CIF3-3HA and KAT80-PTP signals at the new FAZ tip, respectively. Bar, 5 μm. (B) Quantitation of cells with KAT80 localized at the new FAZ tip. One hundred cells of the 2N2K cell type for each cell line were counted, and error bars indicate SD from three independent experimental replicates. ***, *P* < 0.001. (C) Western blotting to examine KAT80 protein stability. KAT80 and CIF3 were endogenously tagged with PTP and triple HA and detected by anti-protein A antibody and anti-HA antibody, respectively. TbPSA6 served as a loading control. The numbers under the KAT80-PTP Western blot indicate the quantitation of the KAT80-PTP band intensity. (D) Effect of KAT80 RNAi on CIF3 localization. Noninduced control and KAT80 RNAi-induced cells (48 h) were used for coimmunostaining. The white arrow and arrowhead indicate KAT80-3HA and PTP-CIF3 signals, respectively. Bar, 5 μm. (E) Quantitation of cells with CIF3 signals at the new FAZ tip. One hundred cells of the 2N2K cell type for each cell line were counted, and error bars indicate SD from three independent experimental replicates. ***, *P* < 0.001. (F) Western blotting to examine CIF3 protein stability. CIF3-PTP and KAT80-3HA were detected by anti-protein A antibody and anti-HA antibody, respectively. TbPSA6 served as a loading control. The numbers under the PTP-CIF3 Western blot indicate the quantitation of the PTP-CIF3 band intensity.

Previous work demonstrated that KAT80 localizes to the new FAZ tip from mitosis until cytokinesis (18), whereas CIF3 localizes to the new FAZ tip from S phase until cytokinesis and to the cleavage furrow during cytokinesis (21). This suggests that CIF3 is recruited to the new FAZ tip earlier than KAT80 and that the two proteins form a complex at late cell cycle stages from mitosis until cytokinesis, during which they colocalize. We tested whether CIF3 is required for KAT80 localization to the new FAZ tip using the CIF3 RNAi cell line expressing endogenously PTP-tagged KAT80 and 3HA-tagged CIF3. Immunofluorescence microscopy confirmed the colocalization of CIF3 and KAT80 at the new FAZ tip in 2N2K cells from the noninduced cell population (Fig. 9A). The depletion of CIF3 by RNAi disrupted the localization of KAT80 to the new FAZ tip, resulting in a reduction of cells with a detectable KAT80 signal at the new FAZ tip from ∼75% to ∼3% of the total 2N2K cell population (Fig. 9A and B). This reduction in KAT80-positive cells was not due to the degradation of KAT80 protein in CIF3 RNAi cells, as demonstrated by Western blotting (Fig. 9C). These results suggest that CIF3 protein is required for KAT80 localization to the new FAZ tip. It was noted that the KAT80 protein level was somewhat increased in CIF3 RNAi cells (Fig. 9C). This was likely due to the increases of 2N2K cells and multinucleated cells and the decrease of 1N1K cells by CIF3 RNAi, as the KAT80 protein level might be lower in G_1_-phase cells than in cells at late cell cycle stages.

Conversely, we tested the effect of KAT80 knockdown on CIF3 localization and stability using the KAT80 RNAi cell line expressing endogenously PTP-tagged CIF3 and 3HA-tagged KAT80. Immunofluorescence microscopy showed that after KAT80 depletion by RNAi, the percentage of 2N2K cells with detectable CIF3 at the new FAZ tip was reduced from ∼89% to ∼54% (Fig. 9D and E). Similarly, this reduction of CIF3-positive cells was not due to any decrease in the CIF3 protein level in KAT80 RNAi cells, as demonstrated by Western blotting (Fig. 9F). These results suggest that KAT80 is required for maintaining CIF3 localization at the new FAZ tip during late cell cycle stages. Similar to the effect of CIF3 RNAi on KAT80 protein (Fig. 9C), the knockdown of KAT80 also caused an increase in the CIF3 protein level after 48 and 72 h (Fig. 9F). This effect is also likely due to the increases of 2N2K cells and multinucleated cells and the decrease of 1N1K cells by KAT80 RNAi (18), as the CIF3 protein level might be lower in G_1_-phase cells than in cells at late cell cycle stages.

**FIG 10:**
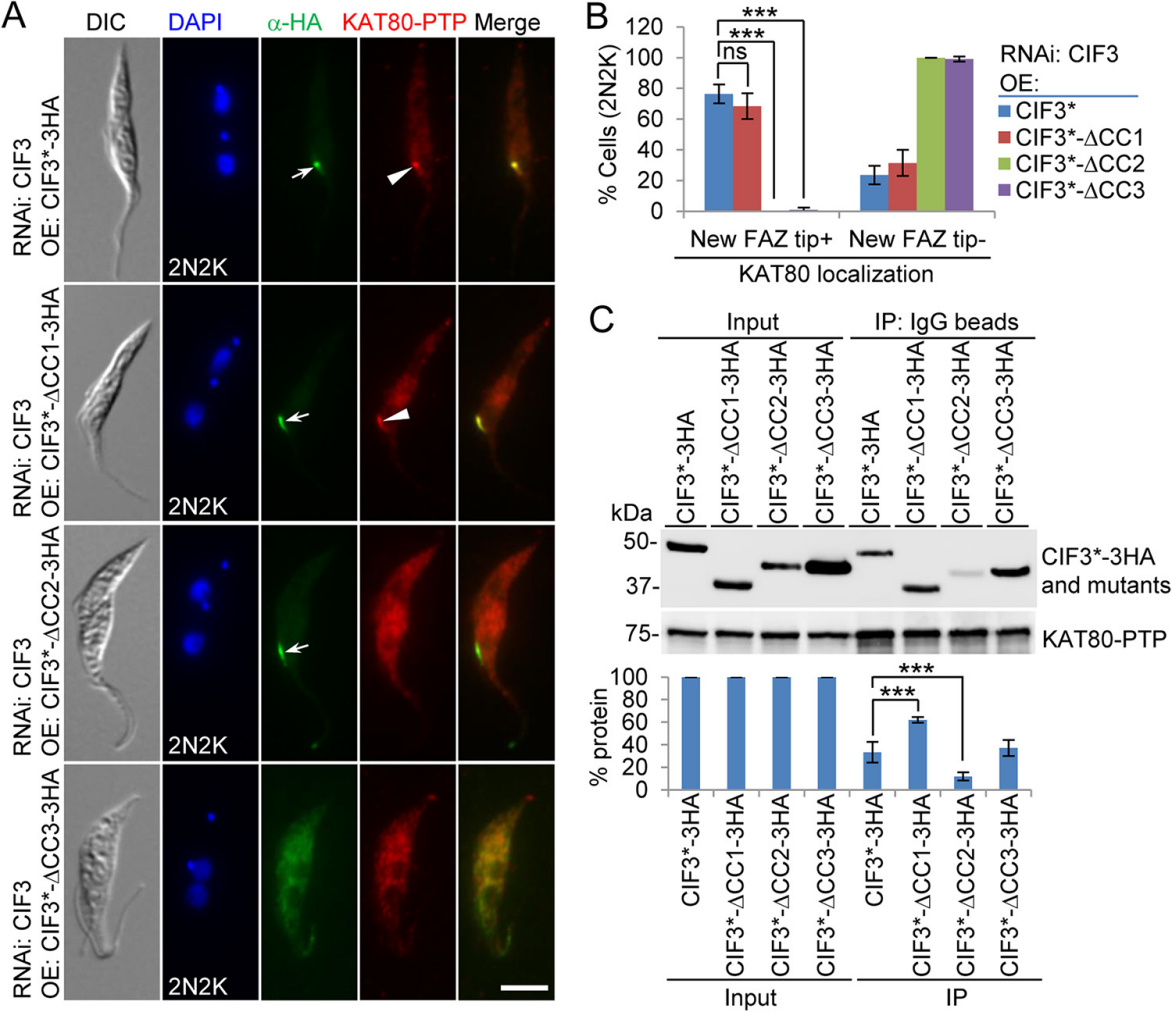
Determination of CIF3 structural motifs required for KAT80 localization to the new FAZ tip. (A) Immunofluorescence microscopy to detect KAT80 localization in CIF3 RNAi complementation cell lines. KAT80 was endogenously tagged with a C-terminal PTP epitope in CIF3 RNAi-induced cells expressing CIF3*-3HA, CIF3*-ΔCC1-3HA, CIF3*-ΔCC2-3HA, or CIF3*-ΔCC3-3HA from an ectopic locus. Cells were induced with tetracycline for 48 h and coimmunostained with anti-HA antibody and anti-protein A antibody. White arrows indicate 3HA-tagged CIF3* and its mutants at the new FAZ tip, whereas white arrowheads indicate KAT80-PTP at the new FAZ tip. Bar, 5 μm. (B) Quantitation of cells with KAT80 localized at the new FAZ tip in CIF3 RNAi cells expressing CIF3*-3HA, CIF3*-ΔCC1-3HA, CIF3*-ΔCC2-3HA, or CIF3*-ΔCC3-3HA after 48 h of tetracycline induction. One hundred cells of the 2N2K cell type for each cell line were counted, and error bars indicate SD from three independent experiments. ***, *P* < 0.001; ns, no significance. (C) Coimmunoprecipitation to test the interaction of CIF3* and its CC deletion mutants with KAT80. KAT80 was endogenously tagged with PTP, immunoprecipitated with IgG beads, and detected by anti-protein A antibody, whereas 3HA-tagged CIF3* and its CC deletion mutants were detected by anti-HA antibody. The graph under the Western blots shows the quantitation of 3HA-tagged CIF3* and its mutants. OE, overexpression.

Using the CIF3 RNAi complementation cell lines expressing endogenously PTP-tagged KAT80, we performed coimmunofluorescence microscopy to examine the requirement of the coiled-coil motifs in CIF3 for the localization of KAT80 and the interaction of CIF3 with KAT80. Immunofluorescence microscopy showed that KAT80 was not detectable at the new FAZ tip in all (100%) of the CIF3 RNAi-induced cells expressing CIF3*-ΔCC2 and in ∼99% of the CIF3 RNAi-induced cells expressing CIF3*-ΔCC3 (Fig. 10A and B). In contrast, KAT80 localization to the new FAZ tip was not significantly affected in the CIF3 RNAi-induced cells expressing CIF3*-ΔCC1, compared to that in the CIF3 RNAi-induced cells expressing CIF3* (Fig. 10A and B). These results suggest that the CC2 and CC3 motifs in CIF3 are each required for KAT80 localization to the new FAZ tip. To test whether the deletion of CC2 or CC3 affected the interaction of CIF3 with KAT80, we performed coimmunoprecipitation experiments. Immunoprecipitation of KAT80-PTP was able to pull down CIF3* and all three CC deletion mutants of CIF3*, but the amounts of coimmunoprecipitated CC deletion mutant CIF3* proteins differed significantly. Compared to the amount of coimmunoprecipitated CIF3*, a significantly larger amount of CIF3*-ΔCC1 was coimmunoprecipitated, a significantly smaller amount of CIF3*-ΔCC2 was coimmunoprecipitated, and a similar amount of CIF3*-ΔCC3 was coimmunoprecipitated (Fig. 10C). This result suggests that the deletion of CC1 strengthens the interaction between CIF3 and KAT80, the deletion of CC2 weakens the interaction between CIF3 and KAT80, and the deletion of CC3 does not affect the interaction between CIF3 and KAT80. Thus, the mislocalization of KAT80 in CIF3 RNAi-induced cells expressing CIF3*-ΔCC2 was apparently attributed to the impaired interaction between CIF3*-ΔCC2 and KAT80, whereas the mislocalization of KAT80 in CIF3 RNAi-induced cells expressing CIF3*-ΔCC3 was attributed to its interaction with the mislocalized CIF3*-ΔCC3 to the cytosol. Altogether, these results demonstrated that CIF3 localization at the new FAZ tip is required for KAT80 localization to the new FAZ tip.

## DISCUSSION

Cytokinesis in the early-branching trypanosomes differs from those of the human host and other eukaryotic organisms by employing an actomyosin-independent mechanism and a trypanosome-specific signaling cascade that acts at the anterior tip of the new FAZ and the ingressing cleavage furrow for cytokinesis initiation and progression. Among the crucial trypanosome-specific cytokinesis regulators discovered so far, CIF1 appears to act as an orchestrator of cytokinesis by interacting and cooperating with almost all of the known cytokinesis regulators, including TbPLK, TbAUK1, CIF2, CIF3, CIF4, FPRC, KLIF, FRW1, KPP1, and KAT80 (18–20). In this work, we provided evidence to demonstrate that CIF3 also plays a pivotal role in regulating cytokinesis by interacting and cooperating with multiple cytokinesis regulators, including TbPLK, CIF1, CIF4, FPRC, and KAT80 but not KLIF and FRW1. Previously, we demonstrated that CIF2 was not an interacting partner of CIF3 (21). Thus, compared to CIF1, CIF3 appears to coordinate the actions of a small set of cytokinesis regulators to promote cytokinesis. It should be noted that the molecular approaches used in this work to test the interaction between CIF3 and other cytokinesis regulators do not demonstrate the interaction to be a direct, physical association. Rather, the interaction between CIF3 and other cytokinesis regulators detected by GST pulldown and co-IP suggests that they form a protein complex through either direct or indirect means. It is also unclear whether CIF3 and its interacting partner proteins form a large protein complex involving three or more subunits, which requires further investigation.

Through genetic complementation, we demonstrated the requirement of coiled-coil motif 2 (CC2) and CC3 for cytokinesis initiation (Fig. 1). However, the mechanisms underlying the cytokinesis defects caused by the deletion of the two CC motifs appeared to be different. In the CIF3*-ΔCC3 complementation cells, CIF3*-ΔCC3 itself and all of the CIF3-interacting cytokinesis regulators were mislocalized from the new FAZ tip to the cytosol, although all proteins but CIF1 maintained binding affinity for CIF3*-ΔCC3 (Fig. 2B and H, Fig. 6C, Fig. 8C, and Fig. 10C). These results suggest that CC3 is necessary for localizing CIF3 to the new FAZ tip, and when CIF3*-ΔCC3 was mislocalized to the cytosol, its binding partners were similarly mislocalized to the cytosol. For those proteins that retained binding affinity for CIF3*-ΔCC3, their mislocalization may be attributed to their binding to the mislocalized CIF3*-ΔCC3. However, for CIF1, which has a weaker binding affinity for CIF3*-ΔCC3 (Fig. 2B), its mislocalization likely was not attributable to its binding to the mislocalized CIF3*-ΔCC3. In the CIF3*-ΔCC2 complementation cells, CIF3*-ΔCC2 itself and all of the CIF3-interacting partner proteins except KAT80 were normally localized to the new FAZ tip (Fig. 3, 6, 8, and 10). Thus, CC2 is not required for CIF3 localization. The underlying mechanism for the cytokinesis defects caused by the deletion of CC2 in CIF3 remains unclear. Although KAT80 was mislocalized to the cytosol in the CIF3*-ΔCC2 complementation cells (Fig. 10), it is unlikely that this contributed to the observed defects in cytokinesis initiation, as KAT80 appears to regulate cytokinesis completion but not cytokinesis initiation (18). Nonetheless, CC2 and CC3 in CIF3 apparently have distinct functions for CIF3’s regulatory roles in cytokinesis initiation. It should be noted that it is possible that the deletion of any of the three CC motifs, in particular CC3, might disrupt the proper folding of CIF3, which may impact the function of CIF3 and the interaction with its partners. However, given that each of the three CC deletion mutants retained binding affinity for various cytokinesis regulators (Fig. 2B and H, Fig. 6C, Fig. 8C, and Fig. 10C), it is possible that the deletion of the CC motifs had little effect on the folding of the CIF3 protein.

We previously demonstrated that CIF1 and CIF3 are functionally interdependent, but the effects exerted by them on each other are different (21). The localization of CIF1 to the new FAZ tip depends on CIF3, whereas the maintenance of the stability of CIF3, but not the localization of CIF3, depends on its interaction with CIF1 (21). Using the CIF3 RNAi complementation cell lines expressing the coiled-coil motif deletion mutants of CIF3, we further confirmed that CIF3 was required for the localization of CIF1 to the new FAZ tip through the interaction with CC3 of CIF3 (Fig. 3A to C). We showed that CC1 in CIF3 also mediated the interaction with CIF1 (Fig. 2A), but the deletion of CC1 exerted no effect on the CIF1-CIF3 interaction (Fig. 2B). Previously, we demonstrated that C-terminal zinc finger motif 1 of CIF1 is required for the interaction with CIF3 (21), and here, we showed that IDR1 of CIF1 also mediated the interaction with CIF3 and that the deletion of IDR1 weakened the interaction between CIF1 and CIF3 (Fig. 2D and E). Together, these results identified the crucial structural motifs of CIF1 and CIF3 required for the formation of the CIF1-CIF3 complex. Moreover, these results also demonstrated that CIF3 played a role in recruiting/targeting CIF1 to the new FAZ tip, suggesting that CIF3 either acts upstream of CIF1 in the cytokinesis regulatory pathway or is recruited/targeted to the new FAZ tip prior to the recruitment of CIF1.

The T. brucei Polo-like kinase homolog TbPLK plays crucial roles in promoting the initiation of cytokinesis in addition to facilitating the duplication of flagellum-associated cytoskeletal structures (14, 15, 29). TbPLK phosphorylates CIF1 and is required for CIF1 localization to the new FAZ tip (19). TbPLK is also required for the formation of the CIF1-CIF3 complex and for the localization of CIF3 to the new FAZ tip after the S phase of the cell cycle (21). We demonstrated here that CIF3 was also a substrate of TbPLK, but mutation of the TbPLK phosphosites in CIF3 did not affect CIF3 function (Fig. 2). This result suggests that CIF3’s function in cytokinesis does not require TbPLK phosphorylation. It appears that the disruption of CIF1-CIF3 complex formation by TbPLK inhibition or knockdown is likely attributable to the impairment of CIF1 phosphorylation and/or localization (19) and that the disruption of CIF3 localization by TbPLK depletion is likely attributable to the failed formation of the CIF1-CIF3 complex (21). In contrast, CIF3 appeared to be required for the localization of TbPLK to the new FAZ tip through interaction with the coiled-coil motifs of CIF3 and the Polo-box domain of TbPLK (Fig. 2 and 3). This role of CIF3 as a TbPLK substrate is in agreement with the finding that most of the PLK substrates discovered in other eukaryotes interact with the Polo-box domain of PLK and are implicated in targeting PLK to various subcellular locations (31).

We previously reported that CIF4 and FPRC interact with CIF1 and demonstrated that both of them are required for CIF1 localization to the new FAZ tip (23) but not vice versa (our unpublished results). CIF4 and FPRC also interact with each other, and CIF4 is required for FPRC localization to the new FAZ tip (23) but not vice versa (our unpublished results). These analyses placed CIF4 upstream of FPRC and placed FPRC upstream of CIF1 in the cytokinesis regulatory pathway. Here, we showed that both CIF4 and FPRC also interacted with CIF3 (Fig. 4) and demonstrated the functional interdependency between CIF3 and its partner proteins CIF4 and FPRC (Fig. 5 to 8). CIF3 was required for targeting CIF4 to the new FAZ tip through interaction with CC1 and CC3 in CIF3 (Fig. 4 and 6), whereas CIF4 appeared to be required for maintaining CIF3 at the new FAZ tip (Fig. 5). The mislocalization of CIF4 to the cytosol in CIF3 RNAi cells expressing the CIF3-ΔCC3 mutant (Fig. 6) suggests that CIF3-ΔCC3 targets CIF4 to the cytosol but not vice versa. Therefore, in wild-type cells, CIF4 apparently acts to maintain, but not target, CIF3 at the new FAZ tip. Similarly, CIF3 also appeared to be required for targeting FPRC to the new FAZ tip through interaction with CC1 and CC3 in CIF3 (Fig. 4 and 8), but FPRC was required for the localization of CIF3 at the new FAZ tip only during late cell cycle stages from anaphase to telophase (Fig. 7). Thus, it appears that CIF3 acts upstream of CIF4 and FPRC in the cytokinesis regulatory pathway or likely is recruited/targeted to the new FAZ tip prior to CIF4 and FPRC.

The katanin subunit KAT80 is the only tested cytokinesis regulator that interacts with all three coiled-coil motifs of CIF3 (Fig. 4). However, only the deletion of coiled-coil motif 2 impaired the interaction between CIF3 and KAT80 (Fig. 10), which caused the mislocalization of KAT80 to the cytosol despite that CIF3-ΔCC2 itself remained to localize to the new FAZ tip (Fig. 10). The deletion of coiled-coil motif 3 also caused the mislocalization of KAT80 to the cytosol (Fig. 10), but CIF3-ΔCC3 itself was similarly mislocalized to the cytosol (Fig. 10). These results suggest that interaction with CIF3 is required for KAT80 localization and that CIF3 acts to recruit KAT80 to the new FAZ tip. Thus, CIF3 functions upstream of KAT80 in the cytokinesis regulatory pathway. This order of action between CIF3 and KAT80 is in agreement with the finding that CIF3 is recruited to the new FAZ tip prior to KAT80 during the cell cycle (18, 21). Interestingly, there appeared to be a feedback effect from KAT80 on CIF3 (Fig. 9D to F), demonstrating the dependence of KAT80 for maintaining CIF3 localization to the new FAZ tip during late cell cycle stages.

**FIG 11:**
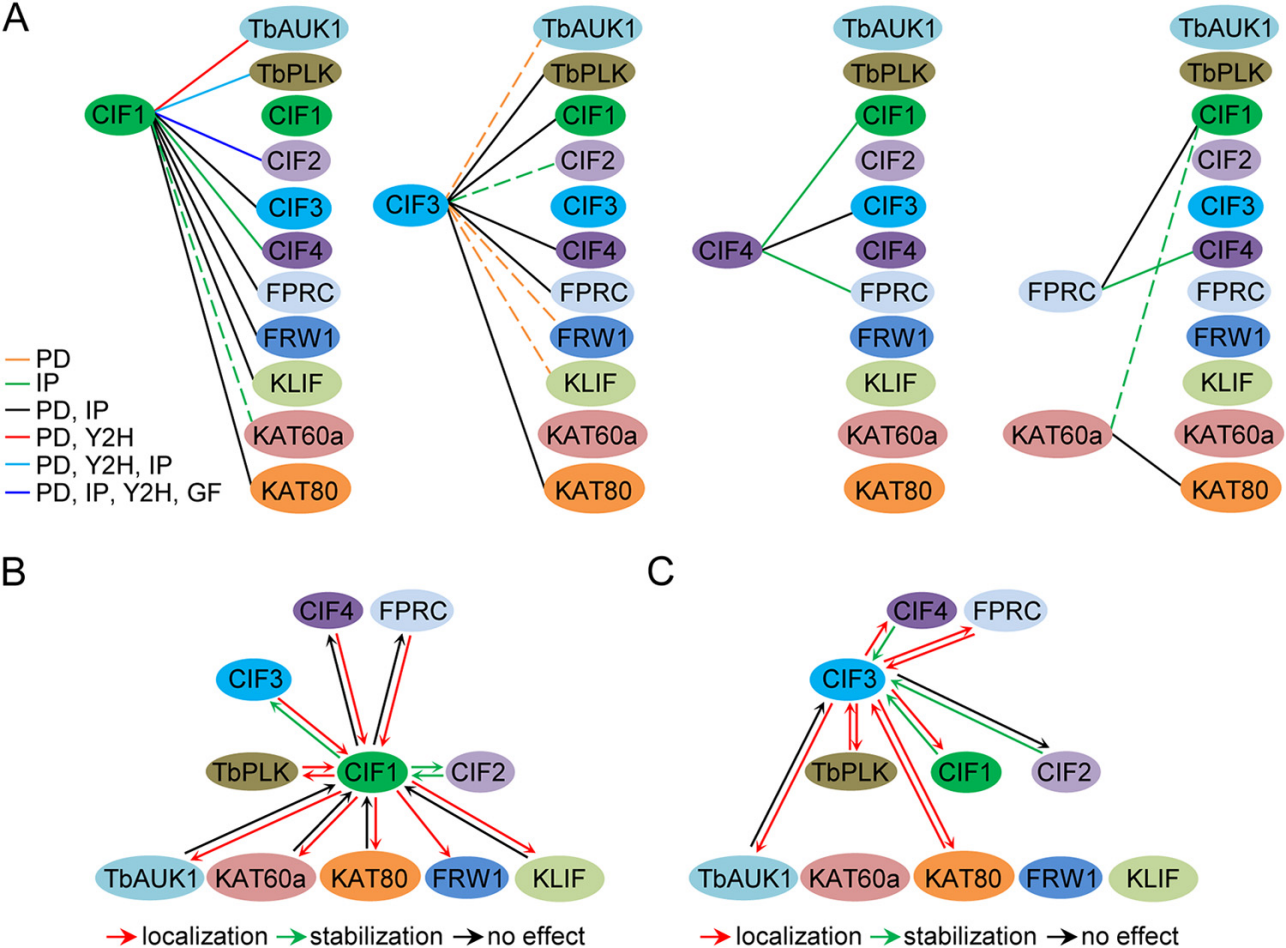
Summary of the interaction and functional interplay among cytokinesis regulators. (A) Interactions among cytokinesis regulators detected by yeast two-hybrid (Y2H), *in vitro* GST pulldown (PD), coimmunoprecipitation (IP), and gel filtration chromatography (GF) assays. The solid line indicates that the interaction was detected by the indicated method(s), whereas the dashed line indicates that the interaction was not detected by the indicated method(s). (B and C). Functional interplay between CIF1 and other cytokinesis regulators (B) and between CIF3 and other cytokinesis regulators (C). Shown are the effects of the knockdown of one regulator on the localization (red arrows) or stability (green arrows) of other regulators. The black arrows indicate that no effect was detected.

Based on the current and previous work performed on the procyclic form of T. brucei, the interaction and the functional interplay among the cytokinesis regulatory proteins are summarized in Fig. 11. Through various approaches, CIF1 was found to interact with 9 out of the 10 cytokinesis regulators, except for KAT60a, which is the catalytic subunit of the microtubule-severing enzyme KAT60a-KAT80 complex (18), whereas CIF3 was found to interact with 5 out of the 9 regulators (Fig. 11A). CIF4 was found to interact with CIF1, CIF3, and FPRC (Fig. 11A), and whether it interacts with other regulators remains to be determined. FPRC was found to interact with CIF1 and CIF4 (Fig. 11A), and whether it interacts with other regulators was not investigated. KAT60a was found to form a complex with KAT80, and it does not interact with CIF1 in co-IP experiments (Fig. 11A) despite that immunoprecipitation of CIF1 was able to pull down KAT80 (18). Furthermore, through genetic and cell biological approaches, the functional interplay among the cytokinesis regulators has been explored (Fig. 11B and C). CIF1 was found to play distinct roles in regulating other regulators. CIF1 maintains the stability of CIF2 and CIF3 and is required for the localization of TbPLK, TbAUK1, KAT60a, KAT80, FRW1, and KLIF but exerts no effect on CIF4 and FPRC (Fig. 11B). Conversely, CIF2 maintains CIF1 stability, and TbPLK, CIF3, CIF4, and FPRC are required for CIF1 localization, but TbAUK1, KAT60a, KAT80, and KLIF exert no effect on CIF1 (Fig. 11B). Note that the effect of FRW1 RNAi on CIF1 was not investigated, but it is very likely that FRW1 exerts no effect on CIF1 due to the lack of any cytokinesis defects by FRW1 RNAi in the procyclic form (18). The investigations on the functional relationship between CIF3 and other cytokinesis regulators demonstrated that CIF3 is required for the localization of TbPLK, TbAUK1, CIF1, CIF4, FPRC, and KAT80 but not for the localization of CIF2 during the S phase of the cell cycle (Fig. 11C), although in the bloodstream form of T. brucei, CIF3 was found to be required for CIF2 localization from S phase to mitosis (25). Conversely, CIF1 and CIF4 are required to maintain CIF3 stability, whereas FPRC and KAT80 are required for the localization of CIF3 (Fig. 11C). However, TbAUK1, which localizes to the new FAZ tip only during late mitosis (16), is not required for CIF3 localization (Fig. 11C). Further exploration of the functional interplay among all of the cytokinesis regulators will provide a comprehensive understanding of how these regulators cooperate to promote cytokinesis in T. brucei.

In summary, we have determined the requirement of the structural motifs in CIF3 for cytokinesis initiation in procyclic trypanosomes, identified the interacting partners of CIF3, and determined the functional interplay between CIF3 and its interacting partner proteins. These results demonstrated a crucial role for CIF3 in recruiting/targeting a cohort of cytokinesis regulatory proteins to the distal tip of the new FAZ to promote cytokinesis initiation and completion and placed CIF3 at the most upstream part of the cytokinesis regulatory pathway discovered in the procyclic form of T. brucei. Finally, our in-depth characterization of the functional interplay between CIF3 and its interacting partner proteins revealed their distinct, cooperative roles in regulating protein localization and stability.

## MATERIALS AND METHODS

### Bioinformatics analysis.

Bioinformatics analysis of CIF3 structural motifs was performed using the DeepCoil algorithm (https://toolkit.tuebingen.mpg.de/tools/deepcoil) (32), the COILS algorithm (https://npsa-prabi.ibcp.fr/cgi-bin/npsa_automat.pl?page=/NPSA/npsa_lupas.html) (33), and hidden Markov models (HMMER) (https://www.ebi.ac.uk/Tools/hmmer/) (34).

### Trypanosome cell culture and RNAi.

T. brucei strain 29-13 (35) was cultured in SDM-79 (Semi-Defined Medium 79) medium supplemented with 10% heat-inactivated fetal bovine serum (Atlanta Biologicals, Inc.), 15 μg/mL G418, and 50 μg/mL hygromycin B at 27°C. The T. brucei strain Lister427 was cultured in SDM-79 medium containing 10% heat-inactivated fetal bovine serum at 27°C. The Lister427 strain expressing endogenously triple-HA-tagged TbAUK1 (19), CIF3 (21), CIF4 (23), FPRC (23), KAT80 (18), FRW1 (18), and KLIF (18) was cultured in SDM-79 medium containing 10% heat-inactivated fetal bovine serum and 1.0 μg/mL puromycin. The CIF3 RNAi cell line (21), the CIF4 RNAi cell line (23), the FPRC RNAi cell line (23), and the KAT80 RNAi cell line (18) were cultured in SDM-79 medium containing 10% heat-inactivated fetal bovine serum and three antibiotics (15 μg/mL G418, 50 μg/mL hygromycin B, and 2.5 μg/mL phleomycin). All these RNAi cell lines were generated using the pZJM vector (36), and hence, they produced double-stranded RNA. Cells were diluted with fresh medium whenever the cell density reached 5 × 10^6^ cells/mL.

### Generation of CIF1 and CIF3 RNAi complementation cell lines.

The CIF1 RNAi complementation cell lines were generated by expressing full-length CIF1, CIF1-ΔIDR1 (Δ1–120), or CIF1-ΔIDR2 (Δ381–651) from an ectopic locus in the CIF1-3′UTR RNAi cell line (26). To this end, the coding sequences of full-length CIF1, CIF1-ΔIDR1, and CIF1-ΔIDR2 were cloned into the pLew100-3HA-BLE vector. The resulting plasmids were each linearized with NotI and electroporated into the CIF1-3′UTR RNAi cell line generated previously (26).

To generate CIF3 RNAi complementation cell lines, an RNAi-resistant CIF3 (designated CIF3*) gene was synthesized (Twist Bioscience), with the codon of the 500-bp DNA fragment (nucleotides 401 to 900), which was used for CIF3 RNAi (21), recoded with a synonymous codon, and cloned into the pLew100-3HA-PAC vector. Subsequently, the DNA fragment encoding CIF3* mutants with a deletion of CC1 (aa 1 to 115), CC2 (aa 175 to 263), or CC3 (aa 364 to 438) was generated and cloned into the pLew100-3HA-PAC vector. Additionally, to generate TbPLK phosphodeficient mutant CIF3*, all five *in vitro* TbPLK phosphosites were mutated to alanine using pLew100-CIF3*-3HA-PAC as the template. These plasmids were each linearized with NotI and electroporated into the CIF3 RNAi cell line generated previously (21).

For both the CIF1 and CIF3 complementation cell lines described above, successful transfectants were selected with 1.0 μg/mL puromycin in addition to 2.5 μg/mL phleomycin, 50 μg/mL hygromycin, and 15 μg/mL G418 and cloned by limiting dilution in a 96-well plate. To induce CIF1 RNAi and the ectopic expression of CIF1-3HA and its mutants and to induce CIF3 RNAi and the ectopic expression of CIF3*-3HA and its various mutants, cells were induced with 1.0 μg/mL tetracycline, and cell growth was monitored daily.

### *In situ* epitope tagging of proteins.

Epitope tagging of proteins from one of their respective endogenous loci was performed by a PCR-based epitope-tagging method (37). CIF3 was endogenously tagged with the PTP epitope at the N terminus in the CIF1-3′UTR RNAi cell line expressing 3HA-tagged CIF1, CIF1-ΔIDR1, or CIF1-ΔIDR2 from an ectopic locus; the CIF4 RNAi cell line expressing CIF4-3HA from its endogenous locus; the CIF3 RNAi cell line expressing 3HA-tagged CIF3* or the CC deletion mutants of CIF3* from an ectopic locus; the FPRC RNAi cell line expressing FPRC-3HA from its endogenous locus; and the KAT80 RNAi cell line expressing KAT80-3HA from its endogenous locus. CIF4 and KAT80 were each tagged with a C-terminal PTP epitope, and FPRC was tagged with an N-terminal PTP epitope in the CIF3 RNAi cell line and the CIF3 RNAi cell line expressing 3HA-tagged CIF3* or the CC deletion mutants of CIF3*. Transfectants were selected with 10 μg/mL blasticidin and cloned by limiting dilution in a 96-well plate.

For coimmunoprecipitation of CIF3 and CIF4, CIF3 and FPRC, and CIF3 and KAT80, CIF3 was endogenously tagged with an N-terminal PTP epitope in T. brucei Lister427 cells expressing CIF4-3HA, FPRC-3HA, or KAT80-3HA from their respective endogenous loci. The transfectants were selected with 1.0 μg/mL puromycin and 10 μ/mL blasticidin and cloned by limiting dilution in a 96-well plate.

### GST pulldown and coimmunoprecipitation.

To express and purify the GST-fused IDR1, CC domain, IDR2, and ZnF domains of CIF1; the three CC domains of CIF3; and the KD and PBD of TbPLK for *in vitro* GST pulldown assays, the DNA sequences encoding the IDR1 (aa 1 to 120), the CC (aa 121 to 271), the IDR2 (aa 272 to 692), and the ZnF (aa 667 to 804) of CIF1; the DNA sequences encoding CC1 (aa 1 to 115), CC2 (aa 175 to 263), and CC3 (aa 363 to 436) of CIF3; and the DNA sequences encoding the KD (aa 1 to 314) and the PBD (aa 434 to 768) of TbPLK were each cloned into the pGEX-4T-3 vector (Clontech). The resultant plasmids were transformed into the E. coli BL21 strain. The expression of the GST-fused proteins was induced with 0.1 mM isopropyl β-d-thiogalactopyranoside for 4 h at room temperature and purified through binding to glutathione-Sepharose 4B beads. GST-fused proteins bound to the beads were incubated with T. brucei lysates prepared from ∼10^8^ cells expressing 3HA-tagged CIF1, TbPLK, TbAUK1, CIF3, CIF4, FPRC, KAT80, FRW1, or KLIF for 30 min at 4°C. The cell lysate was prepared by lysing T. brucei cells in immunoprecipitation (IP) buffer (25 mM Tris-HCl [pH 7.4], 100 mM NaCl, 1 mM dithiothreitol [DTT], 0.07% NP-40, 5% glycerol, and a protease inhibitor cocktail). The beads were washed four times with IP buffer, and bound proteins were eluted by boiling the beads with 1× SDS sampling buffer, separated on SDS-PAGE, transferred onto a polyvinylidene difluoride (PVDF) membrane, and immunoblotted with anti-HA antibody to detect the 3HA-tagged proteins. GST alone was used as the negative control. GST and GST fusion proteins used for pulldown were stained with Coomassie brilliant blue.

Trypanosome cells (∼10^8^) were lysed in 0.5 mL IP buffer for 1 min, and the cell lysate was cleared by centrifugation at 14,000 rpm for 10 min at 4°C in a microcentrifuge. For immunoprecipitation of PTP-tagged proteins (CIF3, CIF4, FPRC, and KAT80), the supernatant was incubated with 25 μL settled IgG-Sepharose beads (GE Healthcare) for 30 min at 4°C. For immunoprecipitation of CIF1 and TbPLK, the supernatant was incubated with 1.0 μL anti-CIF1 antibody (18) or 1.0 μL anti-TbPLK antibody (38) and 15 μL protein A-Sepharose beads for 30 min at 4°C. For immunoprecipitation of 3HA-tagged proteins, the supernatant was incubated with 10 μL settled EZView red anti-HA affinity gel (Sigma-Aldrich) for 30 min at 4°C. Beads were washed five times with IP buffer, and bound proteins were eluted by boiling the beads in 1× SDS-PAGE sampling buffer. Immunoprecipitated proteins were separated by SDS-PAGE, transferred onto a PVDF membrane, and immunoblotted with anti-HA monoclonal antibody (mAb) (clone HA-7, catalog number H9658; Sigma-Aldrich) (1:2,500 dilution) to detect 3HA-tagged proteins, anti-protein A (anti-ProtA) polyclonal antibody (catalog number P3775; Sigma-Aldrich) (1:2,000 dilution) to detect PTP-tagged proteins, anti-CIF1 antibody (1:2,000) to detect CIF1, or anti-TbPLK polyclonal antibody (1:1,000) to detect TbPLK.

The protein band intensity was quantitated using ImageJ software (NIH, Bethesda, MD, USA); normalized with that of CIF1 (Fig. 2B), CIF3 (Fig. 2E), or TbPSA6 (Fig. 9C and F); and then compared with the input (CIF1 and CIF3) (Fig. 2B and E) or the protein level at time zero of RNAi (KAT80 and CIF3) (Fig. 9C and F).

### *In vitro* kinase assay and mass spectrometry.

T. brucei cells expressing TbPLK-3HA were lysed with IP buffer (see above) and cleared by centrifugation. The cleared cell lysate was incubated with EZView red anti-HA affinity gel (Sigma-Aldrich) for 30 min at 4°C, and the beads were washed three times with IP buffer and then two times with kinase assay buffer (10 mM HEPES [pH 7.6], 50 mM NaCl, 10 mM MgCl_2_, 1 mM EGTA, and 1 mM DTT). Immunoprecipitated TbPLK-3HA proteins were incubated with purified recombinant GST-CIF3 in kinase assay buffer with 0.2 mM ATP at room temperature for 30 min. The reaction was stopped by adding 1× SDS sampling buffer, and the beads were boiled for 5 min. Proteins thus eluted were separated by SDS-PAGE and stained with Coomassie brilliant blue. The gel slice containing GST-CIF3 protein was excised for mass spectrometry (MS) to identify phosphopeptides.

In-gel digestion of proteins was carried out as described previously (39). The protein band was digested with 160 ng trypsin (Sigma-Aldrich) at 37°C for 4 h, and peptides were extracted with 50 mL of a solution containing 50% acetonitrile and 5% formic acid. Extracted peptides were dried using a SpeedVac, reconstituted in a solution containing 2% acetonitrile with 0.1% formic acid, and then injected onto a Thermo LTQ Orbitrap XL instrument (Thermo-Fisher Scientific, Bremen, Germany). Samples in 2% (vol/vol) acetonitrile and 0.1% (vol/vol) formic acid were analyzed on an LTQ Orbitrap XL instrument (Thermo-Fisher Scientific) interfaced with an Eksigent nano-liquid chromatography (LC) two-dimensional (2D) plus ChipLC system (Eksigent Technologies, Dublin, CA). The sample was loaded onto a ChromXP C_18_-CL trap column (200-mm internal diameter [ID] by 0.5-mm length) at a flow rate of 3 mL/min. Reversed-phase C_18_ chromatographic separation of peptides was carried out on a ChromXP C_18_-CL column (75-mm ID by 10-cm length) at 300 nL/min. The LTQ Orbitrap was operated in the data-dependent mode to simultaneously measure full-scan MS spectra in the Orbitrap and the five most intense ions in the LTQ by collision-induced dissociation (CID), respectively. In each cycle, MS1 was acquired at a target value of 1E6 with a resolution of 100,000 (*m/z* 400) followed by the top five MS2 scans at a target value of 3E4. The mass spectrometric settings were as follows: the spray voltage was 1.6 kV, and charge state screening and rejection of singly charged ions were enabled. Ion selection thresholds were 8,000 for MS2, 35% normalized collision energy, the activation *Q* value was 0.25, and dynamic exclusion was employed for 30 s. Raw data files were processed and searched against the T. brucei database using the Mascot search engine. The search conditions used a peptide tolerance of 10 ppm and a tandem MS (MS/MS) tolerance of 0.8 Da, with the enzyme set as trypsin and two missed cleavages permitted.

### Immunofluorescence microscopy.

T. brucei cells were adhered to a glass coverslip for 30 min at room temperature, fixed with cold methanol (−20°C) for 30 min, and rehydrated with phosphate-buffered saline (PBS) for 10 min. Cells were then blocked with 3% bovine serum albumin (BSA) in PBS for 30 min at room temperature and incubated with the primary antibody at room temperature for 60 min. For anti-TbPLK polyclonal antibody (1:160 dilution) (38), cells were incubated at 37°C for 1 h. The following primary antibodies were used: anti-CC2D polyclonal antibody (1:2,000 dilution) (7), anti-CIF1 polyclonal antibody (1:1,000 dilution) (18), anti-protein A polyclonal antibody (1:400 dilution) (Sigma-Aldrich), and fluorescein isothiocyanate (FITC)-conjugated anti-HA monoclonal antibody (clone HA-7, catalog number H7411; Sigma-Aldrich) (1:400 dilution). Cells on the coverslip were washed three times with PBS and then incubated with the secondary antibody for 60 min at room temperature. The secondary antibody used is Cy3-conjugated anti-rabbit IgG (catalog number C2306; Sigma-Aldrich) (1:400 dilution). After washing the cells three times with PBS, the slides were mounted in Vectashield mounting medium (Vector Laboratories) containing 4′,6-diamidino-2-phenylindole (DAPI) and examined using an inverted microscope (model IX71; Olympus) equipped with a cooled charge-coupled-device (CCD) camera (model Orca-ER; Hamamatsu) and a Plan Apo N 60× 1.42-numerical-aperture (NA) lens. Images were acquired and processed using Slide-book5 software (Intelligent Imaging Innovations, Inc.).

### Statistical analysis.

Statistical analysis was performed using a chi-square test. Detailed *n* values for each panel in the figures are stated in the corresponding legends. For immunofluorescence microscopy, images were randomly taken, and all cells in each image were counted. Data were collected from three independent experiments.

### Data availability.

All data are contained within the manuscript.
